# Knowledge, attitudes, and practices regarding type 2 diabetes and associated factors among rural adolescents in Indonesia: A cross-sectional study

**DOI:** 10.1371/journal.pone.0352982

**Published:** 2026-07-08

**Authors:** Nila Kusumawati, Hanno Pijl, Halimatou Alaofè, Emul Yani, Desti Puswati, Abdul Hamid

**Affiliations:** 1 Department of Health Promotion Sciences, Mel and Enid Zuckerman College of Public Health, University of Arizona, Tucson, Arizona, United States of America; 2 Indonesian Diabetes Center, Universitas Pahlawan Tuanku Tambusai, Kampar, Riau Province, Indonesia; 3 Department of Internal Medicine, Leiden University Medical Center, Leiden, the Netherlands; 4 Faculty of Nursing, Institut Kesehatan Payung Negeri Pekanbaru, Pekanbaru, Riau Province, Indonesia; The Chinese University of Hong Kong, HONG KONG

## Abstract

**Introduction:**

Type 2 diabetes (T2D) is an increasingly important public health concern in Indonesia and is increasingly affecting adolescents at a growing rate due to lifestyle transitions and limited awareness. Evidence regarding adolescents’ knowledge, attitudes, and practices (KAP) toward T2D in rural settings remains limited. This study assessed KAP levels related to T2D, identified sociodemographic factors associated with KAP outcomes, and examined interrelationships among KAP domains among rural Indonesian adolescents.

**Methods:**

A cross-sectional study was conducted from September 2024 to February 2025 among 1,546 senior high school students in Kampar Regency, Riau Province. Participants were selected using multistage cluster sampling. Data were collected using a validated KAP questionnaire (Cronbach’s α = 0.725). Descriptive and inferential statistics, including Mann–Whitney U tests, Kruskal–Wallis tests, Spearman’s rank correlations, and multiple linear regression analyses, were used to examine KAP outcomes and associated factors.

**Results:**

Participants demonstrated limited knowledge regarding T2D, particularly related to risk factors, symptoms, and complications. Although attitudes toward diabetes prevention were generally positive, preventive practices remained suboptimal, especially regarding physical activity. Female gender, peri-urban school location, higher academic rank, extracurricular participation, and prior exposure to diabetes information were associated with higher KAP scores. Knowledge was moderately associated with attitude but only weakly associated with preventive practices.

**Conclusions:**

Rural adolescents demonstrated limited diabetes-related knowledge and suboptimal preventive behaviors despite generally positive attitudes toward T2D prevention. Several sociodemographic and school-related factors were associated with KAP outcomes. However, the weak relationship between knowledge and practice highlights a persistent gap between awareness and preventive behavior. School-based, peer-led, and after-school interventions that integrate education with behavioral reinforcement may strengthen diabetes prevention efforts among rural adolescents.

## Introduction

Type 2 diabetes (T2D) is a major non-communicable disease in Indonesia, contributing significantly to national morbidity and mortality rates [1–2]. According to the International Diabetes Federation Diabetes Atlas (2025), Indonesia ranks fifth globally in diabetes prevalence among adults aged 20–79 and third for undiagnosed cases. The number of people affected by diabetes and its complications is expected to continue rising [1]. Diabetes is also the fifth leading cause of death among non-communicable diseases in the country [3]. A national study reported that 4% of the 18.9 million enrollees in Indonesia’s National Health Insurance were T2D patients, 57% of whom experienced complications. The direct medical costs of diabetes reached USD 576 million, with 74% of this expenditure dedicated to managing complications [4].

Although T2D was once regarded as an adult-onset disease, it is now increasingly diagnosed among adolescents worldwide [5,6]. Contributing risk factors such as obesity, physical inactivity, and smoking are becoming more prevalent among youth [7]. In rural Indonesia, these risks are compounded by low parental awareness of diabetes screening in adolescents and the widespread perception that T2D primarily affects older adults [8]. The COVID-19 pandemic has further worsened this situation by increasing screen time and reducing physical activity, resulting in higher body mass index (BMI) levels among adolescents [9–11]. Furthermore, the rapid expansion of internet access has contributed to greater digital engagement among adolescents, negatively influencing adolescent lifestyles in both urban and rural areas of Indonesia [12–14].

Reducing adolescents’ exposure to modifiable risk factors is crucial to curbing the future burden of T2D in Indonesia. Evidence suggests that effective prevention programs could reduce national diabetes rates to 9.22% [15]. Designing such interventions requires a clear understanding of adolescents’ knowledge, attitudes, and practices (KAP) related to T2D. Findings from other countries show wide disparities in KAP among adolescent populations. For instance, students in peri-urban areas of Bangladesh demonstrated greater diabetes-related knowledge than those in rural areas [16]; Nepalese metropolitan students showed higher knowledge, but similar attitudes compared to rural peers [17]; and Jordanian adolescents exhibited moderate to high knowledge but poor attitudes and practices [18]. In India, rural high school students displayed fair diabetes knowledge, though their attitudes and self-care behaviors were not assessed [19].

While many studies have examined adolescents’ knowledge and attitudes toward T2D, mainly in urban areas where risk factors are more pronounced, few studies have comprehensively assessed all three KAP domains in rural settings. Even less evidence exists on how sociodemographic factors such as gender, parental education, school type, and extracurricular participation shape these outcomes. Understanding these relationships is crucial for identifying groups at risk of limited awareness or unhealthy behaviors, addressing disparities in access to health information, and designing targeted health promotion strategies. Moreover, most adolescent KAP research in low- and middle-income countries remains descriptive, rarely analyzing interrelationships among KAP domains [16–22]. Exploring these linkages can clarify whether greater knowledge and positive attitudes translate into healthier practices, testing the applicability of the KAP framework.

This study aims to fill these gaps by assessing the levels of KAP related to T2D among rural adolescents in Indonesia; identifying sociodemographic factors associated with these outcomes; and examining the interrelationships among KAP domains. Understanding these relationships is vital for policymakers and educators to design culturally relevant, school-based interventions that promote early diabetes prevention. Strengthening diabetes awareness during adolescence can facilitate early risk identification, encourage healthier lifestyles, and reduce future disease burden. Additionally, this context-specific evidence may inform similar efforts in other low- and middle-income countries, where diabetes prevalence is projected to increase by 95% by 2050 [1]. This project underscores the urgency of developing locally grounded strategies to address the global rise of T2D.

## Materials and methods

### Study design, setting, and period

A cross-sectional study was conducted from September 18, 2024, to February 7, 2025, in Kampar Regency, Riau Province, Indonesia, to assess adolescents’ KAP related to T2D. Riau Province ranks 15th among Indonesia’s 38 provinces in diabetes prevalence, with a rate of 1.5%, slightly below the national average. Unhealthy lifestyle habits, including low physical activity and frequent consumption of sugary beverages, have been observed among children as young as three years old in the region [7,8,12,23]. Kampar Regency was chosen for its predominantly rural characteristics, relatively high diabetes burden, and large population of senior high school students [23].

### Study population, sample size, and sampling

The study population comprised 21,872 senior high school students in Kampar Regency. Using OpenEpi version 3, the minimum sample size was calculated as 1,288 students based on a 50% anticipated frequency, 5% confidence limits, 99% confidence level, and a design effect of 2 to account for cluster sampling. After adjusting for a 20% non-response rate [24], the final sample included 1,546 students to improve statistical power and representativeness.

A multistage cluster sampling technique was used [25]. Eleven of the 21 districts in Kampar were randomly selected, followed by the random selection of one senior high school from each district. Within each school, students in grades 10 and 11 were proportionally recruited. Eligible participants were those attending school during data collection who voluntarily agreed to participate.

### Data collection instrument and procedure

Data were collected using a pretested, structured KAP questionnaire adapted from previous studies on diabetes among adolescents, with additional context-specific variables included to reflect the local setting and study objectives [16–19]. The instrument underwent two stages of validation. First, face validity was assessed by two researchers. Second, content validity was reviewed by six experts in internal medicine, nursing, and public health. This process yielded an item-level content validity index (I-CVI) of 0.80 and a scale-level content validity index (S-CVI) of 0.90.

The questionnaire was pilot-tested among 420 students from three public schools that were not included in the main study. Item validity was supported by Pearson correlation coefficients ranging from 0.273 to 0.585. Construct validity was supported by a Kaiser–Meyer–Olkin (KMO) value of 0.779 and a significant Bartlett’s test of sphericity (p < 0.001). Internal consistency was acceptable, with Cronbach’s alpha of 0.725.

The final instrument, administered in Indonesian (S1 File) and translated into English for reporting purposes (S2 File), consisted of four sections:

#### Sociodemographic and anthropometric characteristics.

The first section assessed sociodemographic and anthropometric characteristics. Sociodemographic variables included age, gender, ethnicity, grade, class rank, school location, total daily school hours, extracurricular participation, parents’ education and employment, and family history of diabetes. Anthropometric measures included height and weight, from which BMI was calculated. Total daily school hours included both regular classroom hours and time spent at school after classes for social, transportation, or educational purposes before returning home. Family history was assessed using two questions: the presence of first-degree relatives with diabetes (none, yes, or no idea), and the number of affected relatives (none or one/more). Height and weight were measured by trained staff to the nearest 0.1 cm and 0.1 kg, respectively. BMI was categorized using the Indonesian classification based on World Health Organization cutoffs [24].

#### Knowledge domain.

The second section assessed knowledge of T2D using five questions covering causes, risk factors, symptoms, complications, and prevention. Some questions allowed multiple responses and were scored based on the number of correct responses selected. Each correct response was assigned 1 point.

#### Attitude domain.

The third section assessed attitudes using six statements related to diabetes prevention and risk factors. Positive responses were scored as 1, and negative or “do not know” responses were scored as 0.

#### Practice domain.

The fourth section assessed practices related to physical activity, smoking, and body weight monitoring. Responses were scored according to the frequency of these health-related behaviors, with higher scores indicating healthier practices. The maximum possible scores were 18 for knowledge, 6 for attitude, and 8 for practice, yielding a total KAP score of 32.

Data were collected in classrooms using self-administered paper questionnaires supervised by the authors and trained assistants from public health, nursing, nutrition, and midwifery programs. Questionnaires were reviewed onsite for completeness before collection, and participants were given the opportunity to review unanswered items during the data collection session.

Data entry was performed by trained research assistants under the supervision of the research team. Prior to data entry, assistants received standardized training on coding procedures, use of the study codebook, and quality control measures. The completed datasets were subsequently cross-checked for accuracy, consistency, and completeness by the research team, and data cleaning was conducted before statistical analysis.

### Ethical considerations

Ethical approval was obtained from the Institutional Review Board of Institut Kesehatan Payung Negeri Pekanbaru, Riau Province, Indonesia (No. 277/IKES PN/KEPK/IX/2024). The study was conducted in accordance with the approved protocol, and no major deviations occurred during the study period. Permission to conduct data collection in schools was granted by the Provincial Department of Education (No.421/cabdisdik/6.2/2023/083), and additional written approvals were obtained from each participating school.

Participation was voluntary, and all participants were informed about the study’s purpose and procedures. Participants were informed that they could decline participation or withdraw from the study at any time without penalty. Written informed consent was obtained from students aged 17 years and older, witnessed by homeroom teachers. For students under 17 years, written parental or guardian consent and participant assent were required.

Questionnaires were anonymous, and no identifying information was collected. Completed questionnaires were coded and stored securely in a locked cabinet, while electronic data files were stored on password-protected personal computers. Both hard-copy and electronic data were accessible only to the research team and retained for up to three years after study completion. Data were analyzed and reported in aggregate form to maintain participant confidentiality.

### Inclusivity in global research and reporting guidelines

Additional information regarding the ethical, cultural, and scientific considerations specific to inclusivity in global research is provided in the Supporting Information (S3 File). This study was also reported in accordance with the Strengthening the Reporting of Observational Studies in Epidemiology (STROBE) guidelines. The completed STROBE checklist is provided in the Supporting Information (S1 Checklist).

### Statistical analysis

Descriptive statistics were used to summarize participants’ sociodemographic characteristics and KAP outcomes. Categorical variables were presented as frequencies (n) and percentages (%), while continuous variables with non-normal distributions were summarized using medians and interquartile ranges (IQRs). There were no missing data in the dataset.

For descriptive interpretation, individual knowledge, attitude, practice, and total KAP scores were converted into percentages of the maximum obtainable scores and categorized using modified Bloom’s cutoff criteria as low/negative (<60%), moderate/neutral (60%–79%), or high/positive (≥80%) [26]. These categories were used solely to describe KAP levels, while continuous KAP scores were retained for bivariate and multivariable analyses.

The Shapiro–Wilk test indicated that KAP scores were not normally distributed (p < 0.001); therefore, non-parametric tests were used for bivariate analyses. Spearman’s rank correlation was used to examine associations between age and KAP scores. The Mann–Whitney U test was used for comparisons between binary variables, such as gender and school location, while the Kruskal–Wallis test was used for variables with three or more categories, such as family history of T2D.

Multiple linear regression analyses were subsequently performed to identify sociodemographic predictors of KAP scores, with the composite scores treated as continuous outcomes. Linear regression was considered appropriate because the composite KAP scores were derived from multiple questionnaire items and demonstrated sufficient range and approximate continuity. In addition, regression diagnostics indicated that model assumptions were adequately met at the residual level. All measured sociodemographic variables were included simultaneously to adjust for potential confounding. Although multistage cluster sampling was used, analyses were conducted at the individual level.

Regression assumptions were evaluated systematically. Linearity assumptions were satisfied based on diagnostic assessment. Visual inspection of residual plots suggested approximate normality of residuals. Residual normality was considered acceptable for linear regression, particularly given the large sample size. Heteroskedasticity was detected using White’s test (p < 0.05) and was addressed using robust standard errors. Independence of observations was considered satisfied based on the study design. No multicollinearity was detected, with a mean variance inflation factor (VIF) of 1.17. No influential observations were identified based on Cook’s Distance (Cook’s D < 0.05). Diagnostic tests, including the Ramsey RESET and Link tests, showed no evidence of model misspecification (p > 0.05).

Finally, Spearman’s rank correlation was used to examine interrelationships among the KAP domains. All analyses were conducted using Stata Statistical Software, Release 19 (StataCorp LLC, College Station, TX, USA). A two-sided p-value < 0.05 was considered statistically significant.

## Results

### Participants’ sociodemographic characteristics

A total of 1,546 senior high school students participated in the study, with a median age of 16 years (IQR: 16–17). As shown in Table 1, nearly two-thirds of participants were female (64.0%), and the majority were non-obese (91.8%). Most students attended peri-urban schools (63.8%), spent more than 8 hours/day at school (93.3%), and did not participate in extracurricular activities (65.8%).

**Table 1:** Sociodemographic characteristics of the study participants (N = 1,546).

Characteristics	n (%)
**Gender**	
Male	557 (36.0)
Female	989 (64.0)
**Ethnicity**	
Indigenous	834 (53.9)
Non-Indigenous	712 (46.1)
**Body Mass Index**	
Obese	126 (8.2)
Non-obese	1,420 (91.8)
**School Location**	
Remote rural	559 (36.2)
Peri-urban	987 (63.8)
**Total Daily School Hours**	
≤8 hours/day	103 (6.7)
>8 hours/day	1,443 (93.3)
**Grade Level**	
Grade X	716 (46.3)
Grade XI	830 (53.7)
**Class Rank**	
Below top 10	942 (60.9)
Top 10	604 (39.1)
**Extracurricular Participation**	
Not participating	1,017 (65.8)
Participating at least 1 day/week	529 (34.2)
**Father’s Employment**	
Unemployed	114 (7.4)
Employed	1,432 (92.6)
**Mother’s Employment**	
Unemployed	1,258 (81.4)
Employed	288 (18.6)
**Father’s Education**	
Junior high school or below	583 (37.7)
Senior high school or above	963 (62.3)
**Mother’s Education**	
Junior high school or below	596 (38.6)
Senior high school or above	950 (61.4)
**First-degree Relatives’ T2D History**	
None	1,098 (71.0)
Yes	141 (9.1)
No idea	307 (19.9)
**Received Information about T2D**	
Did not receive	364 (23.5)
Received	1,182 (76.5)

Regarding parental characteristics, 62.3% of fathers and 61.4% of mothers had at least a senior high school education. Fathers were more likely to be employed (92.6%) than mothers (18.6%). Almost 20% of adolescents were uncertain whether their first-degree relatives had T2D. Among participants reporting a family history of T2D, all reported at least one affected first-degree relative, most commonly their father (57.4%). Additionally, 23.5% of participants had not received any information about diabetes. Among those who had received information about diabetes, social media (48.8%), websites (42.3%), and school-based education (26.1%) were the most common information sources.

### Knowledge of type 2 diabetes

As shown in Table 2, participants demonstrated partial knowledge of T2D. About 16.6% incorrectly believed it to be an infectious disease, while 22.4% were uncertain. Only 50.8% recognized physical inactivity as a risk factor, and 8.9% identified smoking as a risk factor. Awareness of common symptoms was also limited. While 53.0% recognized frequent urination, only 16.9% identified increased thirst and 16.2% identified frequent hunger. Knowledge of T2D complications varied. While 61.5% of participants identified foot problems as a complication, only 13.0% recognized diabetic retinopathy. Regarding preventability, 81.6% correctly indicated that T2D is preventable, although 15.7% were unsure and 2.7% believed it was not preventable.

**Table 2:** Participants’ diabetes-related knowledge (N = 1,546).

Questions and Selected Responses	n	%
**T2D is an infectious disease**		
Yes	256	16.6
No	943	61.0
Do not know	347	22.4
**T2D risk factors**		
Obesity	603	39.0
Physical inactivity	785	50.8
Family history	814	52.7
Stress	140	9.1
Smoking	137	8.9
**T2D signs and symptoms**		
Frequent urination	819	53.0
Tiredness	570	36.9
Increased thirst	262	16.9
Increased hunger	250	16.2
Unintended weight loss	543	35.1
**T2D complications**		
Diabetic retinopathy	201	13.0
Diabetic kidney disease	434	28.1
Foot problems	951	61.5
Nerve damage	323	20.9
Heart disease	229	14.8
Stroke	207	13.4
**T2D is preventable**		
Yes	1,262	81.6
No	42	2.7
Do not know	242	15.7

### Attitudes toward type 2 diabetes

Participants’ attitudes toward diabetes prevention and T2D risk factors are presented in Table 3. Most adolescents expressed positive attitudes toward prevention, with 91.1% agreeing that healthy eating is important and 89.1% acknowledging the role of exercise in T2D prevention. However, misconceptions persisted. Nearly 28% were unsure whether having a family history increases T2D risk, and 27.7% disagreed. Likewise, 24.4% disagreed that smoking raises the risk, and 43.0% were uncertain. Approximately one-quarter (24.8%) of participants were unsure of the role of obesity in increasing T2D risk.

**Table 3:** Participants’ diabetes-related attitudes (N = 1,546).

Questions and Selected Responses	n	%
**Healthy eating is important to prevent T2D**		
Agree	1,408	91.1
Disagree	19	1.2
Not sure	119	7.7
**Exercise helps prevent T2D**		
Agree	1,378	89.1
Disagree	30	1.9
Not sure	138	8.9
**If you have a family history of T2D, you are at higher risk of getting it**		
Agree	677	43.8
Disagree	428	27.7
Not sure	441	28.5
**People who smoke are more likely to get T2D**		
Agree	503	32.6
Disagree	377	24.4
Not sure	655	43.0
**Regular screening helps in the early detection and prevention of T2D**		
Agree	1,273	82.3
Disagree	43	2.8
Not sure	230	14.9
**Obesity increases the risk of developing T2D**		
Agree	1,034	66.9
Disagree	129	8.3
Not sure	383	24.8

### Preventive practices related to type 2 diabetes

Preventive practices related to T2D varied across behaviors (Table 4). Only 17.5% of adolescents engaged in at least 60 minutes of daily physical activity, whereas 66.0% were active for 30 minutes or less per day, and 16.5% reported no physical activity. Although 91.5% of participants were non-smokers, 6.4% reported smoking fewer than 25 cigarettes per day, and 2.1% reported smoking 25 or more cigarettes per day. Regarding weight monitoring, only 5.0% checked their weight daily, whereas 16.0% did so annually, and 3.8% never monitored their weight.

**Table 4:** Participants’ diabetes-related practices (N = 1,546).

Questions and Selected Responses	n	%
**Daily Physical Activity**		
None	255	16.5
30 minutes or less	1021	66.0
60 minutes or more	270	17.5
**Cigarette Consumption per Day**		
Never	1,414	91.5
Less than 25	99	6.4
25 or more	33	2.1
**Frequency of Body Weight Monitoring**		
Never	58	3.8
Once a year	248	16.0
Once every few months	540	34.9
Once a month	404	26.1
Once a week	219	14.2
Every day	77	5.0

### Overall knowledge, attitude, and practice levels

The median (IQR) knowledge score was 5 (4–7) out of a possible score of 18, with most participants (92.4%) classified as having low knowledge levels regarding T2D. The median (IQR) attitude score was 4 (3–5) out of a possible score of 6, and 42.9% of participants demonstrated positive attitudes toward T2D prevention. The median (IQR) practice score was 4 (3–5) out of a possible score of 8, with more than half of participants (55.5%) categorized as having low practice levels. The median overall KAP score was 14 (12–17) out of a possible score of 32, and most participants (89.1%) demonstrated low overall KAP levels related to T2D (Table 5).

**Table 5:** Distribution of knowledge, attitude, practice, and overall KAP levels (N = 1,546).

Domain	Low n (%)	Moderate n (%)	High n (%)
Knowledge	1,429 (92.4)	102 (6.6)	15 (1.0)
Attitude	459 (29.7)	423 (27.4)	664 (42.9)
Practice	858 (55.5)	585 (37.8)	103 (6.7)
Total KAP^a^	1,377 (89.1)	158 (10.2)	11 (0.7)

^a^KAP: knowledge, attitude, and practice.

KAP levels were categorized using modified Bloom’s cutoff criteria as low (<60%), moderate (60%–79%), or high (≥80%).

### Factors associated with knowledge, attitude, and practice

#### Bivariate analysis.

Bivariate analyses (S1 Table) revealed significant associations between several sociodemographic factors and KAP domains. Gender, school location, grade level, extracurricular participation, parental education, and exposure to diabetes information were significantly related to knowledge, attitude, and overall KAP scores (all p < 0.05), but not to practice scores. Longer school hours and higher class rank were significantly associated with multiple KAP domains, whereas obesity status and parental employment showed no significant associations. Participants reporting a family history of T2D had higher attitude and overall KAP scores than those without such history or who were uncertain about their family history status. Adolescents who were uncertain about their family’s T2D status generally exhibited the lowest KAP scores across domains.

#### Multivariable analysis of sociodemographic factors.

After adjustment for all covariates, several sociodemographic variables remained significantly associated with knowledge scores.

#### Knowledge.

Female students scored, on average, approximately 0.5 points higher on the knowledge scale than male students (B = 0.50, 95% CI 0.24–0.77, p < 0.001). Non-Indigenous students also demonstrated higher knowledge scores than Indigenous students (B = 0.80, 95% CI 0.53–1.07, p < 0.001). Higher knowledge scores were additionally observed among students attending peri-urban schools (B = 0.46, 95% CI 0.17–0.74, p = 0.002), those in Grade XI (B = 0.33, 95% CI 0.02–0.64, p = 0.040), students ranked in the top 10 of their class (B = 0.55, 95% CI 0.27–0.83, p < 0.001), participants in extracurricular activities (B = 0.72, 95% CI 0.43–1.00, p < 0.001), students whose fathers had at least a senior high school education (B = 0.34, 95% CI 0.05–0.63, p = 0.020), and those previously exposed to diabetes-related information (B = 0.48, 95% CI 0.20–0.75, p = 0.001). In contrast, students who were uncertain about their family’s diabetes history demonstrated lower knowledge scores (B = –0.36, 95% CI –0.66 to –0.05, p = 0.020).

#### Attitude.

Higher attitude scores were observed among female students (B = 0.35, 95% CI 0.20–0.50, p < 0.001), peri-urban school attendees (B = 0.29, 95% CI 0.15–0.43, p < 0.001), students spending more than 8 hours/day at school (B = 0.51, 95% CI 0.21–0.81, p = 0.001), those in Grade XI (B = 0.21, 95% CI 0.04–0.37, p = 0.013), and students ranked in the top 10 of their class (B = 0.20, 95% CI 0.06–0.35, p = 0.005). Students participating in extracurricular activities (B = 0.24, 95% CI 0.10–0.39, p = 0.001) and those previously exposed to diabetes-related information (B = 0.34, 95% CI 0.17–0.52, p < 0.001) also demonstrated more favorable attitude scores toward T2D prevention and risk factors. Conversely, students uncertain about their family’s diabetes history demonstrated lower attitude scores (B = –0.20, 95% CI –0.38 to –0.01, p = 0.040).

#### Practice.

Higher practice scores were observed among students attending peri-urban schools (B = 0.16, 95% CI 0.02–0.30, p = 0.030), those spending more than 8 hours per day at school (B = 0.69, 95% CI 0.43–0.94, p < 0.001), and students ranked in the top 10 of their class (B = 0.24, 95% CI 0.10–0.38, p = 0.001). Students uncertain about their family’s diabetes history again demonstrated lower practice scores (B = –0.18, 95% CI –0.34 to –0.01, p = 0.040).

#### Overall KAP.

Higher overall KAP scores were observed among female (B = 0.88, 95% CI 0.49–1.26, p < 0.001) and non-Indigenous students (B = 0.93, 95% CI 0.55–1.30, p < 0.001), as well as among students attending peri-urban schools (B = 0.90, 95% CI 0.50–1.30, p < 0.001), those spending more than 8 hours per day at school (B = 1.26, 95% CI 0.41–2.11, p = 0.004), students in higher grade level (B = 0.54, 95% CI 0.10–0.98, p = 0.015), those ranked in the top 10 of their class (B = 0.99, 95% CI 0.60–1.39, p < 0.001), and participants in extracurricular activities (B = 1.01, 95% CI 0.62–1.40, p < 0.001). Higher paternal education (B = 0.45, 95% CI 0.04–0.86, p = 0.03) and prior exposure to diabetes-related information (B = 0.85, 95% CI 0.43–1.26, p < 0.001) were also associated with higher overall KAP scores. In contrast, adolescents uncertain about their family’s diabetes history consistently demonstrated lower overall KAP scores (B = –0.73, 95% CI –1.18 to –0.28, p = 0.002).

Although the observed effect sizes were generally modest in magnitude, several associations were consistently identified across the knowledge, attitude, practice, and overall KAP domains. Given the multifactorial nature of health knowledge and behaviors, even relatively small differences in KAP scores across adolescent populations may be meaningful at the population level, particularly for informing targeted diabetes education and prevention strategies among vulnerable adolescent groups (Table 6).

**Table 6:** Associations between sociodemographic characteristics and KAP scores (N = 1,546).

Variables	Knowledge	Attitude	Practice	Overall KAP
**B (95% CI),** ***p***	**B (95% CI),** ***p***	**B (95% CI),** ***p***	**B (95% CI),** ***p***
**Age**	0.02 (–0.17, 0.22), 0.81	0.05 (–0.06, 0.16), 0.38	–0.02 (–0.12, 0.08), 0.72	0.06 (–0.22, 0.34), 0.69
**Gender**				
Male	Ref	Ref	Ref	Ref
Female	0.50 (0.24, 0.77), < 0.001	0.35 (0.20, 0.50), < 0.001	0.03 (–0.12, 0.17), 0.71	0.88 (0.49, 1.26), < 0.001
**Ethnicity**				
Indigenous	Ref	Ref	Ref	Ref
Non-Indigenous	0.80 (0.53, 1.07), < 0.001	0.14 (0.00, 0.28), 0.06	–0.01 (–0.15, 0.12), 0.85	0.93 (0.55, 1.30), < 0.001
**Body mass index**				
Obese	Ref	Ref	Ref	Ref
Non-obese	–0.21 (–0.65, 0.24), 0.37	–0.10 (–0.33, 0.12), 0.38	–0.22 (–0.46, 0.02), 0.08	–0.53 (–1.17, 0.12), 0.11
**School location**				
Remote rural	Ref	Ref	Ref	Ref
Peri-urban	0.46 (0.17, 0.74), 0.002	0.29 (0.15, 0.43), <0.001	0.16 (0.02, 0.30), 0.03	0.90 (0.50, 1.30), <0.001
**Total daily school hours**				
≤8 hours	Ref	Ref	Ref	Ref
>8 hours	0.06 (–0.51, 0.64), 0.83	0.51 (0.21, 0.81), <0.001	0.69 (0.43, 0.94), <0.001	1.26 (0.41, 2.11), 0.004
**Grade level**				
Grade X	Ref	Ref	Ref	Ref
Grade XI	0.33 (0.02, 0.64), 0.04	0.21 (0.04, 0.37), 0.01	0.01 (–0.16, 0.17), 0.95	0.54 (0.10, 0.98), 0.02
**Class rank**				
Below top 10	Ref	Ref	Ref	Ref
Top 10	0.55 (0.27, 0.83), <0.001	0.20 (0.06, 0.35), 0.01	0.24 (0.10, 0.38), <0.001	0.99 (0.60, 1.39), <0.001
**Extracurricular participation**				
Not participating	Ref	Ref	Ref	Ref
Participating at least 1 day/week	0.72 (0.43, 1.00), <0.001	0.24 (0.10, 0.39), 0.001	0.05 (–0.09, 0.19), 0.48	1.01 (0.62, 1.40), <0.001
**Father’s education**				
Junior high school or below	Ref	Ref	Ref	Ref
Senior high school or above	0.34 (0.05, 0.63), 0.02	0.08 (–0.08, 0.24), 0.34	0.03 (–0.12, 0.18), 0.72	0.45 (0.04, 0.86), 0.03
**Mother’s education**				
Junior high school or below	Ref	Ref	Ref	Ref
Senior high school or above	0.00 (–0.29, 0.30), 0.98	0.08 (–0.09, 0.24), 0.36	0.04 (–0.11, 0.20), 0.58	0.12 (–0.30, 0.54), 0.56
**Father’s employment**				
Unemployed	Ref	Ref	Ref	Ref
Employed	0.18 (–0.27, 0.64), 0.43	0.07 (–0.21, 0.35), 0.62	–0.11 (–0.37, 0.16), 0.43	0.15 (–0.53, 0.83), 0.67
**Mother’s employment**				
Unemployed	Ref	Ref	Ref	Ref
Employed	0.08 (–0.26, 0.42), 0.65	–0.05 (–0.23, 0.12), 0.55	–0.07 (–0.25, 0.11), 0.44	–0.04 (–0.53, 0.45), 0.87
**First-degree relatives with T2D**^**a**^ **history**				
None	Ref	Ref	Ref	Ref
Yes	0.19 (–0.27, 0.65), 0.41	0.19 (–0.03, 0.41), 0.08	0.03 (–0.22, 0.27), 0.84	0.41 (–0.22, 1.04), 0.20
No idea	–0.36 (–0.66, –0.05), 0.02	–0.20 (–0.38, –0.01), 0.04	–0.18 (–0.34, –0.01), 0.04	–0.73 (–1.18, –0.28), 0.001
**Received information about T2D**				
Not received	Ref	Ref	Ref	Ref
Received	0.48 (0.20, 0.75), 0.001	0.34 (0.17, 0.52), <0.001	0.03 (–0.13, 0.19), 0.73	0.85 (0.43, 1.26), <0.001

Values are presented as regression coefficients (B) with 95% confidence intervals.

Reference categories are indicated as “Ref”.

a T2D: Type 2 diabetes.

Model fit statistics: Knowledge, F(16,1529) = 13.19, p < 0.001, R² = 0.110; Attitude, F(16,1529) = 9.77, p < 0.001, R² = 0.093; Practice, F(16,1529) = 3.68, p < 0.001, R² = 0.034; Overall KAP, F(16,1529) = 16.36, p < 0.001, R² = 0.143.

### Interrelationships among KAP domains

As shown in Table 7, Spearman’s rank correlation analysis demonstrated significant positive associations among KAP domains (all p < 0.05). Knowledge was moderately correlated with attitude (ρ = 0.424), whereas correlations between knowledge and practice (ρ = 0.052) and between attitude and practice (ρ = 0.117) were weak. Knowledge, attitude, and practice scores were all positively correlated with overall KAP scores, with the strongest correlation observed between knowledge and overall KAP (ρ = 0.817). These findings suggest that although knowledge and attitudes toward T2D were positively related, their associations with preventive practices were relatively weak.

**Table 7:** Intercorrelations among KAP domains.

KAP Domains	Spearman’s ρ (p-value)
Knowledge – Attitude	0.424 (<0.001)
Knowledge – Practice	0.052 (0.040)
Attitude – Practice	0.117 (<0.001)
Knowledge – Overall KAP	0.817 (<0.001)
Attitude – Overall KAP	0.706 (<0.001)
Practice – Overall KAP	0.438 (<0.001)

## Discussion

This study examined KAP related to T2D among rural Indonesian adolescents. It also identified sociodemographic factors associated with KAP outcomes and explored interrelationships among the domains. The findings revealed generally low levels of diabetes-related knowledge and suboptimal engagement in preventive practices among adolescents. Although some participants demonstrated favorable attitudes toward T2D prevention, overall KAP levels remained low. These findings suggest that positive attitudes alone may not be sufficient to promote healthy preventive behaviors in the absence of adequate knowledge and supportive environments for behavioral change. They also underscore the need for school-based interventions that strengthen diabetes-related understanding while promoting sustainable behavioral change among adolescents.

### Knowledge and attitudes toward T2D

Adolescents demonstrated limited knowledge of T2D, particularly regarding smoking as a risk factor and symptoms such as excessive thirst and hunger. Similar knowledge gaps have been reported among adolescents and school students in rural India, Nepal, Bangladesh, Kuwait, and Saudi Arabia, especially regarding T2D risk factors and symptoms [16,17,19,27,28]. Although most participants recognized that T2D is preventable, many remained uncertain about its causes and complications. Additionally, nearly one-quarter of participants had never received any information about diabetes, and school-based education was reported less frequently than social media and websites as a source of diabetes-related information. These findings suggest that existing school-based health education may not provide sufficient depth or interactive learning opportunities to support comprehensive diabetes-related understanding.

Despite these knowledge gaps, many participants expressed favorable attitudes toward diabetes prevention and acknowledged the importance of healthy eating and physical activity. This finding is consistent with studies from rural Nepal and Bangladesh, where adolescents reported positive attitudes despite relatively limited knowledge levels [16,17]. These positive attitudes may serve as an important foundation for future diabetes prevention efforts among rural adolescents. However, misconceptions regarding smoking and family history as risk factors indicate that some adolescents still hold inaccurate or inconsistent beliefs about T2D causation. Such misconceptions may hinder the translation of positive attitudes into effective preventive behaviors.

### Knowledge and preventive practices

This study revealed low levels of both diabetes-related knowledge and preventive practices among adolescents. In addition, the correlation between knowledge and practice was the weakest among the KAP domain relationships. Although approximately half of participants recognized physical inactivity as a risk factor for T2D, only 17.5% met the recommended 60 minutes of daily physical activity. Similar findings have been reported in Nepal, Ethiopia, and Indonesia, where physical inactivity among adolescents remains common [20,29,30].

These findings suggest that awareness alone is insufficient to support behavioral change and that adolescent health behaviors are shaped by broader environmental and sociocultural influences. Low physical activity levels may be influenced by hot weather conditions, limited peer and parental support for active lifestyles, and gender-related preferences to avoid outdoor activity [20,29,30]. Adolescents may also view T2D primarily as a disease affecting older adults, potentially reducing their perceived susceptibility and motivation to engage in preventive behaviors [8]. Although smoking prevalence was relatively low, adolescents who smoked tended to consume relatively large numbers of cigarettes, possibly reflecting the continued accessibility and social acceptability of tobacco use among Indonesian youth [31]. Together, these factors may hinder the adoption of healthy preventive behaviors despite some awareness of diabetes prevention practices.

These findings highlight the need for multifaceted interventions that go beyond information provision. Health promotion efforts should also strengthen behavioral skills, motivation, and supportive social environments. Strategies such as experiential learning, goal setting, and peer modeling may help reinforce the link between awareness and healthy behaviors among adolescents.

### Influence of sociodemographic factors

Sociodemographic characteristics significantly influenced KAP outcomes. Female students consistently demonstrated higher knowledge, attitudes, and overall KAP scores, consistent with findings from Jordan, Iraq, and urban Indonesia showing that female adolescents often exhibit greater health literacy and attentiveness to health-related issues [18,21,22]. In rural settings, these differences may additionally reflect gender-related variations in socialization, communication preferences, and exposure to health information. Educational interventions should therefore adopt gender-responsive approaches and engage male adolescents through activity-based and technology-driven programs. These findings highlight the importance of considering sociodemographic context when designing adolescent diabetes prevention programs, particularly within rural settings where access to health education and preventive resources may vary.

Students attending peri-urban schools and those in higher grades demonstrated greater knowledge and more positive attitudes toward T2D. These differences may reflect greater cognitive maturity, better access to information, and increased exposure to structured health education [17,21]. Longer school hours and higher academic achievement were also associated with healthier preventive practices. This finding suggests that greater academic engagement and self-discipline may support greater health awareness and behavioral regulation [32–33]. Together, these findings highlight schools as critical platforms for structured, evidence-based health promotion.

Participation in extracurricular activities such as sports, scouting (Pramuka), and student councils was associated with higher knowledge, attitude, and overall KAP scores. These activities may provide opportunities for peer interaction, leadership development, and informal learning that reinforce health awareness [28,34]. Integrating diabetes prevention messages into existing extracurricular programs and encouraging greater participation among students in rural areas may therefore improve the reach and sustainability of health promotion efforts.

Adolescents who were uncertain about their family’s diabetes history consistently demonstrated lower KAP scores across domains. This finding may reflect lower perceived susceptibility to T2D and limited family communication regarding diabetes risk. Encouraging communication between adolescents and families regarding diabetes and family health history may help improve awareness and motivation for preventive behaviors.

Exposure to diabetes-related information through social media, digital platforms, or school programs was also linked to higher KAP scores, highlighting the potential role of digital communication in supporting adolescent health literacy [16,21,22]. Leveraging social media and school-supported digital health campaigns may help expand the reach of diabetes education in resource-limited rural settings, provided that the content is accurate, engaging, and culturally relevant.

### Interrelationships among KAP domains

Although knowledge and attitude scores were positively correlated with overall KAP scores, their relationships with preventive practices were weak. Similar findings have been reported in Jordan, Nepal, and Iraq [17,18,22]. These findings suggest that the traditional KAP framework in which knowledge and attitudes are expected to support healthy behaviors may not fully capture the complexity of adolescent preventive practices. Adolescent health behaviors are also influenced by psychosocial and environmental determinants, including peer influence, family norms, social support, and the increasing independence of adolescents in shaping their own health behaviors [34].

Integrating broader behavioral frameworks, such as the Health Belief Model and Social Cognitive Theory, may strengthen diabetes education by addressing motivational, social, and contextual influences alongside cognitive learning. From a Health Belief Model perspective, adolescents may possess some basic diabetes-related awareness yet still lack sufficient perceived susceptibility or cues to action to adopt preventive behaviors. Social Cognitive Theory further suggests that adolescent behaviors are shaped not only by knowledge, but also by peer modeling, family influences, social reinforcement, and self-efficacy. These frameworks therefore help explain why awareness and positive attitudes did not consistently translate into healthier preventive practices in this study.

### Implications for practice and policy

These findings carry important practical and policy implications for diabetes prevention among adolescents in rural Indonesia. Schools are strategic settings for health promotion because of their broad reach and structured environments. School-based interventions should be tailored to the limited resources and sociocultural contexts of rural communities. Rather than relying solely on classroom-based instruction, diabetes prevention strategies may be more sustainable when integrated into existing school and community structures, including extracurricular activities, school health units (Usaha Kesehatan Sekolah/UKS), peer-led initiatives, and collaborations with primary health centers (Puskesmas) and community health cadres. Participatory approaches such as group discussions, peer mentoring, and activity-based learning may further improve student engagement and knowledge retention.

Given the increasing use of smartphones among adolescents, including those in rural areas, low-cost digital approaches such as digital health education programs, short educational videos, and social media campaigns may further expand access to diabetes-related information in remote communities. Investments in teacher training, school health infrastructure, and digital learning resources are also important to support long-term program sustainability. Policymakers should additionally address broader structural and environmental barriers, including challenges related to engaging in physical activity and the accessibility of tobacco products, which may undermine health promotion efforts in rural communities.

### Strengths and limitations

This study contributes to the limited evidence on diabetes-related KAP among adolescents in rural Asia. Its large school-based sample and analysis of sociodemographic correlates strengthen the relevance of the findings for similar rural contexts. Furthermore, examining interrelationships among KAP domains provides additional insight into the relationship between awareness and behavior.

However, several limitations should be noted. The cross-sectional design precludes causal inference, and the use of self-reported data may have introduced social desirability and recall bias, particularly regarding family history of T2D and prior exposure to diabetes-related information. Selection bias may also have occurred because only students enrolled in school and present during data collection were included. In addition, the quantitative nature of the study may not have fully captured the complexity of adolescents’ beliefs or contextual influences on behavior. Finally, the practice domain focused primarily on physical activity, smoking, and weight monitoring, while other relevant behaviors such as dietary habits, screen time, and sleep patterns were not assessed and may also influence adolescents’ risk of T2D.

## Conclusion

Rural Indonesian adolescents demonstrated limited knowledge and suboptimal preventive behaviors related to T2D despite relatively favorable attitudes toward prevention. Sociodemographic and school-related factors, particularly gender, academic performance, school location, and extracurricular participation, were associated with more favorable KAP outcomes. Longer school hours and higher academic achievement were also associated with healthier preventive practices. Weak correlations between preventive practices and the knowledge and attitude domains highlight a gap between diabetes-related awareness and preventive behaviors among adolescents. Addressing this gap requires school-based, peer-led, and after-school interventions, such as extracurricular activities, that integrate behavioral reinforcement into health education. Strengthening diabetes literacy and promoting healthy lifestyle behaviors during adolescence may help reduce early T2D risk and mitigate the long-term burden of diabetes in Indonesia and other low- and middle-income settings.

## Supporting information

S1 File Questionnaire in Indonesian language. (DOCX)

S2 File Questionnaire in English language. (DOCX)

S3 File Inclusivity in global research questionnaire. (DOCX)

S1 Checklist STROBE Checklist. (DOC)

S1 Table Results of the bivariate analyses. (DOCX)
