# Supplementary material for: Knowledge, attitudes, and practices regarding type 2 diabetes and associated factors among rural adolescents in Indonesia: A cross-sectional study
Source: PLoS One. 2026 Jul 8;21(7):e0352982. doi: 10.1371/journal.pone.0352982 (PMC13345253; doi:10.1371/journal.pone.0352982)
Supplement: S1 File — (DOCX) [file pone.0352982.s001.docx]

**Kuesioner**Pengetahuan, Sikap, dan Perilaku Siswa/Siswi SMA Negeri dalam Pencegahan Penyakit Diabetes Melitus Tipe 2

**Petunjuk Pengisian**

- Silakan membaca setiap pertanyaan dengan teliti sebelum menjawab.
- Tidak ada jawaban benar atau salah. Jawablah sesuai dengan pengetahuan, pendapat, dan perilaku anda.
- Seluruh informasi yang diberikan akan dijaga kerahasiaannya dan hanya digunakan untuk kepentingan penelitian.
- Untuk pertanyaan dengan kotak centang (☐), beri tanda centang (✓) pada jawaban yang paling sesuai dengan kondisi anda.
- Beberapa pertanyaan hanya dapat memilih satu jawaban, sedangkan pertanyaan lainnya dapat memilih lebih dari satu jawaban.
- Pertanyaan yang memperbolehkan lebih dari satu jawaban akan diberi keterangan “Anda bisa memilih beberapa pilihan.”
- Jika terdapat pertanyaan yang kurang dipahami, anda dapat bertanya kepada peneliti.

**Informasi Sosiodemografis**

Umur: __________________ tahun

Jenis Kelamin: ☐ Laki-laki ☐ Perempuan

Sekolah: ________________________________________________

Kelas: _________________________________________________

Ranking belajar di kelas: ☐ Masuk 10 besar ☐ Tidak masuk 10 besar

Suku: ______________________________________________

Tinggi Badan: __________ cm

Berat Badan: __________ kg

Berapa hari anda belajar di sekolah dalam satu minggu? ________________________

Berapa jam anda belajar di sekolah dalam satu hari? ______________________

Jika anda mengikuti ekstrakurikuler, tuliskan hari apa saja anda mengikutinya: ____________________

Pekerjaan Ayah: ____________________________________________

Pekerjaan Ibu: ____________________________________________

Pendidikan Ayah: _____________________________________________

Pendidikan Ibu: _____________________________________________

**1. Apakah ada anggota keluarga anda (ibu, ayah, atau saudara kandung) yang memiliki riwayat penyakit diabetes?**

☐ Ya ☐ Tidak ☐ Tidak tahu

(*Jika menjawab tidak/tidak tahu, lanjut ke pertanyaan nomor 3)

**2. Hubungan anda dengan anggota keluarga yang mengalami diabetes (Anda bisa memilih lebih dari satu pilihan):**

☐ Ibu kandung ☐ Ayah kandung ☐ Saudara laki-laki ☐ Saudara perempuan

**3. Apakah anda pernah mendapatkan informasi terkait diabetes?**

☐ Pernah ☐ Tidak pernah

(*Jika menjawab tidak pernah, lanjut ke pertanyaan nomor 5)

**4. Jika pernah mendengar diabetes, dari mana anda mendapatkan informasi terkait kesehatan? (Anda bisa memilih beberapa pilihan):**

☐ Sekolah ☐ Televisi ☐ Website ☐ Media sosial
☐ Radio ☐ Koran ☐ dan lain-lain: __________

**Pengetahuan tentang Diabetes**

**5. Apakah diabetes merupakan penyakit menular?**

☐ Ya ☐ Tidak ☐ Tidak tahu

**6. Menurut anda, apa saja penyebab penyakit diabetes? (Anda bisa memilih beberapa pilihan)**

☐ Kegemukan ☐ Kurang aktivitas fisik ☐ Riwayat keluarga diabetes
☐ Stres ☐ Merokok

**7. Menurut anda, apa saja gejala diabetes? (Anda bisa memilih beberapa pilihan)**

☐ Sering buang air kecil ☐ Selalu merasa lelah ☐ Sering merasa haus
☐ Sering merasa lapar ☐ Berat badan menurun tanpa sebab yang jelas

**8. Di bawah ini adalah komplikasi diabetes (Anda bisa memilih beberapa opsi):**

☐ Kerusakan pada retina mata ☐ Gangguan ginjal ☐ Luka pada kaki
☐ Kerusakan pada syaraf ☐ Serangan jantung ☐ Stroke

**9. Apakah diabetes dapat dicegah?**

☐ Ya ☐ Tidak ☐ Tidak tahu

**Sikap tentang Diabetes**

| 10. Diabetes dapat dicegah dengan menjaga pola makan. | ☐ Setuju | ☐ Tidak setuju | ☐ Tidak tahu |
| --- | --- | --- | --- |
| 11. Olahraga teratur dapat mencegah diabetes. | ☐ Setuju | ☐ Tidak setuju | ☐ Tidak tahu |
| 12. Jika anggota keluarga atau saudara sedarah anda menderita diabetes, anda juga berisiko terkena diabetes. | ☐ Setuju | ☐ Tidak setuju | ☐ Tidak tahu |
| 13. Merokok dapat menyebabkan diabetes. | ☐ Setuju | ☐ Tidak setuju | ☐ Tidak tahu |
| 14. Pemantauan gula darah secara teratur membantu mengendalikan diabetes. | ☐ Setuju | ☐ Tidak setuju | ☐ Tidak tahu |
| 15. Kegemukan dapat menyebabkan penyakit diabetes. | ☐ Setuju | ☐ Tidak setuju | ☐ Tidak tahu |

**Perilaku tentang Diabetes**

**16. Seberapa sering anda berolahraga?**

| ☐ Tidak pernah | ☐ ≤ 30 menit per hari | ☐ Minimal 60 menit per hari |
| --- | --- | --- |

**17. Seberapa sering anda merokok?**

| ☐ Tidak pernah | ☐ Kurang dari 25 batang rokok per hari | ☐ 25 batang rokok atau lebih per hari |
| --- | --- | --- |

**18. Seberapa sering anda menimbang berat badan?**

| ☐ Setiap hari | ☐ Sekali seminggu | ☐ Sekali sebulan |
| --- | --- | --- |
| ☐ Sekali dalam beberapa bulan | ☐ Sekali setahun | ☐ Tidak pernah |

**Terima kasih atas partisipasi anda!**

| **Code** |  |
| --- | --- |
