# Supplementary material for: Knowledge, attitudes, and practices regarding type 2 diabetes and associated factors among rural adolescents in Indonesia: A cross-sectional study
Source: PLoS One. 2026 Jul 8;21(7):e0352982. doi: 10.1371/journal.pone.0352982 (PMC13345253; doi:10.1371/journal.pone.0352982)
Supplement: S2 File — (DOCX) [file pone.0352982.s002.docx]

**Questionnaire**Knowledge, Attitudes, and Practices of Senior High School Students on the Prevention of Type 2 Diabetes Mellitus

**Instructions for Participants**

- Please read each question carefully before answering.
- There are no right or wrong answers. Please answer honestly based on your own knowledge, opinions, and behaviors.
- All information provided will be kept confidential and used only for research purposes.
- For questions with checkboxes (☐), place a check mark (✓) in the box that best represents your answer.
- Some questions allow only one answer, while others allow multiple answers.
- Questions allowing multiple answers are marked with “You may choose multiple answers.”
- If you do not understand a question, you may ask the researcher for clarification.

**Sociodemographic Information**

Age: __________________ years

Gender: ☐ Male ☐ Female

School: ________________________________________________

Grade: _________________________________________________

Class ranking: ☐ Within the top 10 ☐ Not within the top 10

Ethnicity: ______________________________________________

Height: __________ cm Weight: __________ kg

How many days a week do you attend school? ________________________

How many hours a day do you study at school? ______________________

If you participate in extracurricular activities, please state on which days: ____________________

Father’s occupation: ____________________________________________

Mother’s occupation: ____________________________________________

Father’s education: _____________________________________________

Mother’s education: _____________________________________________

**1. Does any member of your family (mother, father, or sibling) have a history of diabetes?**

☐ Yes ☐ No ☐ Don’t know

(*If “No” or “Don’t know,” continue to question 3)

**2. Your relationship with the family member who has diabetes (You may choose more than one):**

☐ Mother ☐ Father ☐ Brother ☐ Sister

**3. Have you ever received any information about diabetes?**

☐ Yes ☐ No

(*If “No,” skip to question 5)

**4. If yes, where did you get information about diabetes? (You may choose multiple sources):**

☐ School ☐ Television ☐ Website ☐ Social Media
☐ Radio ☐ Newspaper ☐ Others: __________

**Knowledge about Diabetes**

**5. Is diabetes an infectious disease?**

☐ Yes ☐ No ☐ Don’t know

**6. According to you, what are the causes of diabetes? (You may choose multiple answers)**

☐ Obesity ☐ Lack of physical activity ☐ Family history of diabetes
☐ Stress ☐ Smoking

**7. What are the symptoms of diabetes? (You may choose multiple answers)**

☐ Frequent urination ☐ Constant fatigue ☐ Frequent thirst
☐ Frequent hunger ☐ Unexplained weight loss

**8. Below are complications of diabetes (You may choose multiple answers):**

☐ Eye (retina) disease ☐ Kidney problems ☐ Foot ulcers
☐ Nerve damage ☐ Heart attack ☐ Stroke

**9. Can diabetes be prevented?**

☐ Yes ☐ No ☐ Don’t know

**Attitudes about Diabetes**

| 10. Diabetes can be prevented by maintaining a healthy diet. | ☐ Agree | ☐ Disagree | ☐ Don’t know |
| --- | --- | --- | --- |
| 11. Regular exercise can prevent diabetes. | ☐ Agree | ☐ Disagree | ☐ Don’t know |
| 12. If your family member or blood relative has diabetes, you are also at risk. | ☐ Agree | ☐ Disagree | ☐ Don’t know |
| 13. Smoking can cause diabetes. | ☐ Agree | ☐ Disagree | ☐ Don’t know |
| 14. Regular blood sugar monitoring helps control diabetes. | ☐ Agree | ☐ Disagree | ☐ Don’t know |
| 15. Obesity can cause diabetes. | ☐ Agree | ☐ Disagree | ☐ Don’t know |

# Practices about Diabetes

**16. How often do you exercise?**

| ☐ Never | ☐ ≤ 30 minutes/day | ☐ At least 60 minutes/day |
| --- | --- | --- |

**17. How often do you smoke?**

| ☐ Never | ☐ Fewer than 25 cigarettes/day | ☐ 25 cigarettes or more/day |
| --- | --- | --- |

**18. How often do you weigh yourself?**

| ☐ Every day | ☐ Once a week | ☐ Once a month |
| --- | --- | --- |
| ☐ Once every few months | ☐ Once a year | ☐ Never |

| **Code** |  |
| --- | --- |

**Thank you for your participation!**
