# Supplementary material for: Knowledge, attitudes, and practices regarding type 2 diabetes and associated factors among rural adolescents in Indonesia: A cross-sectional study
Source: PLoS One. 2026 Jul 8;21(7):e0352982. doi: 10.1371/journal.pone.0352982 (PMC13345253; doi:10.1371/journal.pone.0352982)
Supplement: S3 File — (DOCX) [file pone.0352982.s003.docx]

Inclusivity in global research

PLOS’ policy on inclusivity in global research aims to improve transparency in the reporting of research performed outside of researchers’ own country or community and ensures that PLOS publications reporting global research adhere to high standards for research ethics and authorship. Authors of relevant research articles may be asked to complete the questionnaire below, which outlines ethical, cultural, and scientific considerations specific to inclusivity in global research. This questionnaire may be requested when researchers have travelled to a different country to conduct research, if research uses samples collected in another country, research with Indigenous populations or their lands, or if research is on cultural artefacts. Researchers travelling to another country solely to use laboratory equipment will not normally be required to complete the questionnaire. However, the questionnaire can be requested at the journal’s discretion for any submission – if you have been requested to complete this questionnaire by the PLOS journal you submitted to, please do so.

Please complete the questionnaire below and include this as a Supporting Information file with your manuscript. Note that if your paper is accepted for publication, this checklist will be published with your article in the supporting information files. Please ensure that you reference the checklist in the main body of your manuscript. We suggest adding a subsection ‘Inclusivity in global research’ to your Methods section and adding the following sentence: “Additional information regarding the ethical, cultural, and scientific considerations specific to inclusivity in global research is included in the Supporting Information (SX Checklist)”

The questions have been designed to be applicable to a wide range of study types, and there are subsections for both human subjects research and non-human subjects research. If any of the questions are not relevant to your research please mark them as “N/A” as appropriate.

**Ethical considerations, permits and authorship**

*This section is applicable to all research types.*

Provide details as to who granted permissions and/or consent for the study to take place in the Methods section of your manuscript. This should include the names of **all** ethics boards, governmental organizations, community leaders or other bodies that provided approval for the study. If individuals provided approval refer to these people by their role or title but do not list their name(s).

Ethical approval was obtained from the Institutional Review Board of Institut Kesehatan Payung Negeri Pekanbaru, Riau Province, Indonesia (No. 277/IKES PN/KEPK/IX/2024). The study was conducted in accordance with the approved protocol, and no major deviations occurred during the study period. Permission to conduct data collection in schools was granted by the Provincial Department of Education (No.421/cabdisdik/6.2/2023/083), and additional written approvals were obtained from the principals of each participating school.

If there were any deviations from the study protocol after approval was obtained please provide details of these changes in the Methods section of your manuscript.

The study was conducted in accordance with the approved protocol, and no major deviations occurred during the study period.

Did this study involve local collaborators that are residents of the country where the research was conducted or members of the community studied? If you do not have any authors from said communities, please provide an explanation for this below.

Everyone listed as an author should meet PLOS’ criteria for authorship and all individuals who meet these criteria should be included in the author byline, rather than the acknowledgements. For further information please see the journal’s Authorship Policy.

Yes. Authors 1, 4, 5, and 6 are Indonesian researchers and were residents of the study region during the conduct of the study. Data collection was carried out while these authors were physically based in the study area. The study further involved collaboration with local schools and the Provincial Department of Education.

**Human subjects research (e.g. health research, medical research, cross-cultural psychology)**

Did you obtain written informed consent from a representative of the local community or region before the research took place? How did you establish who speaks for the community? Details of written informed consent obtained from study participants should be reported separately in the Methods section of your manuscript.

Yes. Prior to data collection, permission for school-based data collection was obtained from the Provincial Department of Education in Riau Province, Indonesia, and additional written approvals were obtained from each participating school. These institutions were considered the appropriate authorities to provide authorization for research conducted in school settings because they oversee educational administration and student-related activities within the study region. Written informed consent procedures for individual participants are described separately in the Methods section of the manuscript. In addition, Written informed consent procedures for individual participants were conducted separately. Written informed consent was obtained from students aged 17 years and older, witnessed by homeroom teachers. For students under 17 years, written parental or guardian consent and participant assent were required.

How did members of the local community provide input on the aims of the research investigation, its methodology, and its anticipated outcome(s)?

Input from local stakeholders was incorporated primarily through collaboration with local educational authorities and participating schools during study planning and implementation. The study addressed a locally relevant public health concern regarding adolescents’ knowledge, attitudes, and practices related to type 2 diabetes in Kampar Regency, Indonesia.

The questionnaire was adapted to the Indonesian context using evidence from previous studies and underwent face and content validity assessment by Indonesian researchers and experts in public health, nursing, and internal medicine. In addition, the instrument was pilot-tested among local students prior to the main study to assess clarity, cultural appropriateness, and feasibility. Feedback obtained during these processes informed refinement of the questionnaire and study procedures.

When engaging with the local community, how did you ensure that the informed consent documents and other materials could be understood by local stakeholders?

All informed consent documents and study materials were prepared in the Indonesian language, the primary language of the study population, to facilitate comprehension among participants and local stakeholders. Participants and parents/guardians were encouraged to ask questions regarding any unclear information, and the research team provided verbal explanations and clarification before written consent or assent was obtained.

Will the findings of the research be made available in an understandable format to stakeholders in the community where the study was conducted (e.g. via a presentation, summary report, copies of publications, etc.)? Please provide details of how this will be achieved.

Yes. The findings of the study will be made available to relevant local stakeholders, including participating schools and the Provincial Department of Education, through summary reports and copies of the published article where appropriate. Findings may also be communicated through academic presentations and educational discussions with the Department of Health of Kampar Regency, Riau Province, where the study was conducted, as well as with school health services, school principals, and primary health care centers overseeing the participating schools. Efforts will be made to use accessible language to facilitate understanding and potential application in adolescent health promotion programs.

**Non-human subjects research using specimens/ animals collected as part of the study, or those housed in archival collections. Examples include archaeology, paleontology, botany and zoology.**

Did the permission you obtained from a local authority to perform the study include an agreement on access to outputs and benefit sharing? This may include procedures to enable fair distribution of the benefits and resources arising from the research performed. Please include any details of Prior Informed Consent and Benefit Sharing Agreements obtained. These may be required by field-specific regulations, for example the Convention on Biological Diversity (CBD) and the associated Nagoya Protocol.

Not applicable. This study did not involve non-human specimens, biological materials, animals, archaeological materials, or genetic resources requiring benefit-sharing agreements or permits under the Convention on Biological Diversity (CBD) or the Nagoya Protocol.

If the material used in your study was imported, please A) provide the year it was imported and B) indicate whether permits were obtained to import/export the materials used, C) provide details of any permits obtained. If this information is not available, please indicate this.

Not applicable. This study did not involve the import or export of biological specimens, materials, or other regulated research resources requiring import/export permits.

If you used archival specimens, please state how the material used in your study was acquired by the institute it is held in and provide details of any permits obtained for the original excavations/ sample collection. If this information is not available, please indicate this.

Not applicable. This study did not involve archival specimens, biological samples, archaeological materials, or museum collections.

How was the potential cultural significance of the materials collected in your study to local communities considered in your research design? Were Indigenous peoples and/or local researchers and institutions involved with archaeological excavations / collection of specimens? If so, please provide a description of their involvement.

Not applicable. This study did not involve archaeological excavations, specimen collection, cultural artifacts, biological materials, or Indigenous heritage materials. The study consisted solely of questionnaire-based data collection among students in school settings.

If your manuscript includes photographs of human remains please indicate whether authors obtained permission from descendants or affiliated cultural communities to do so.

Not applicable. This manuscript does not include photographs of human remains.
