# Supplementary material for: Knowledge, attitudes, and practices regarding type 2 diabetes and associated factors among rural adolescents in Indonesia: A cross-sectional study
Source: PLoS One. 2026 Jul 8;21(7):e0352982. doi: 10.1371/journal.pone.0352982 (PMC13345253; doi:10.1371/journal.pone.0352982)
Supplement: S1 Table — (DOCX) [file pone.0352982.s005.docx]

**S1 Table. Results of the bivariate analyses.**

**Comparison of KAP scores across participants’ sociodemographic characteristics.**

| **Variables** | **Knowledge** | | **Attitude** | | **Practice** | | **KAP** | |
| --- | --- | --- | --- | --- | --- | --- | --- | --- |
|  | **Median (IQR)** | **ρ*/ z ** / χ²***, p** | **Median (IQR)** | **ρ*/ z ** / χ²***, p** | **Median (IQR)** | **ρ*/ z ** / χ²***, p** | **Median (IQR)** | **ρ*/ z ** / χ²***, p** |
| **Gender**** | | | | | | | | |
| Male | 5 (4–7) | –5.4, <0.001 | 4 (3–5) | –5.71, <0.001 | 4 (3–5) | –1.13, 0.26 | 13 (11–16) | –6.22, <0.001 |
| Female | 6 (4–8) |  | 4 (3–5) |  | 4 (4–5) |  | 15 (12–17) |  |
| **Ethnicity** ** | | | | | | | | |
| Indigenous | 5 (4–7) | –7.61, <0.001 | 4 (3–5) | –3.59, 0.003 | 4 (3–5) | –1.49, 0.14 | 14 (12–16) | –6.78, <0.001 |
| Non-Indigenous | 6 (5–8) |  | 4 (3–5) |  | 4 (4–5) |  | 15 (13–18) |  |
| **Body Mass Index** ** | | | | | | | | |
| Obese | 6 (4–8) | 1.42, 0.16 | 4 (3–5) | 0.78, 0.44 | 4 (4–6) | 1.69, 0.09 | 15 (12–18) | 1.72, 0.086 |
| Non-obese | 5 (4–7) |  | 4 (3–5) |  | 4 (3–5) |  | 14 (12–17) |  |
| **School Location** ** | | | | | | | | |
| Remote rural area | 5 (4–7) | –4.48, <0.001 | 4 (3–5) | –4.44, <0.001 | 4 (3–5) | –1.87, 0.06 | 14 (12–16) | –5.11, <0.001 |
| Peri-urban area | 6 (5–8) |  | 4 (4–5) |  | 4 (4–5) |  | 15 (13–18) |  |
| **Total Daily School Hours** ** | | | | | | | | |
| ≤8 hours | 5 (4–7) | –2.32, 0.02 | 4 (3–5) | –3.52, <0.001 | 4 (3–4) | –5.03, <0.001 | 12 (10–16) | –4.13, <0.001 |
| >8 hours | 5 (4–7) |  | 4 (3–5) |  | 4 (4–5) |  | 14 (12–17) |  |
| **Grade Level** ** | | | | | | | | |
| Grade X | 5 (4–7) | –4.21, <0.001 | 4 (3–5) | –3.89, <0.001 | 4 (3–5) | 0.11, 0.92 | 14 (12–16) | –3.74, <0.001 |
| Grade XI | 5 (4–8) |  | 4 (3–5) |  | 4 (3–5) |  | 14 (12–17) |  |
| **Class Rank**** | | | | | | | | |
| Below top 10 | 5 (4–7) | –6.96, <0.001 | 4 (3–5) | –4.61, <0.001 | 4 (3–5) | –2.90, 0.004 | 14 (12–16) | –7.40, <0.001 |
| Top 10 | 6 (5–8) |  | 4 (4–5) |  | 4 (4–5) |  | 15 (13–18) |  |
| **Extracurricular Participation**** | | | | | | | | |
| Not participating | 5 (4–7) | –5.88, <0.001 | 4 (3–5) | –3.54, <0.001 | 4 (3–5) | –0.78, 0.44 | 14 (12–16) | –5.93, <0.001 |
| Participating at least 1 day/week | 6 (5–9) |  | 4 (3–5) |  | 4 (4–5) |  | 15 (13–18) |  |
| **Father’s Employment**** | | | | | | | | |
| Non-employed | 5 (4–7) | –1.31, 0.19 | 4 (3–5) | –0.71, 0.48 | 4.5 (3–5) | 0.77, 0.45 | 14 (12–17) | –0.76, 0.45 |
| Employed | 5 (4–7) |  | 4 (3–5) |  | 4 (3–5) |  | 14 (12–17) |  |
| **Mother’s Employment**** | | | | | | | | |
| Unemployed | 5 (4–7) | –0.04, 0.97 | 4 (3–5) | 0.21, 0.84 | 4 (4–5) | 0.61, 0.54 | 14 (12–17) | 0.02, 0.99 |
| Employed | 5 (4–8) |  | 4 (3–5) |  | 4 (3–5) |  | 14 (12–17) |  |
| **Father’s Education**** | | | | | | | | |
| Junior high school or below | 5 (4–7) | –4.13, <0.001 | 4 (3–5) | –2.90, 0.004 | 4 (3–5) | –1.15, 0.25 | 14 (12–16) | –4.32, <0.001 |
| Senior high school or above | 5 (4–8) |  | 4 (3–5) |  | 4 (4–5) |  | 15 (12–17) |  |
| **Mother’s Education**** | | | | | | | | |
| Junior high school or below | 5 (4–7) | –2.94, 0.003 | 4 (3–5) | –2.50, 0.013 | 4 (3–5) | –0.91, 0.37 | 14 (12–16) | –3.35, <0.001 |
| Senior high school or above | 5 (4–8) |  | 4 (3–5) |  | 4 (4–5) |  | 15 (12–17) |  |
| **First-degree Relatives’ T2D History***** | | | | | | | | |
| None | 5 (4–8) | 10.06, 0.007 | 4 (3–5) | 12.24, 0.002 | 4 (4–5) | 6.64, 0.04 | 14 (12–17) | 18.11, <0.001 |
| Yes | 5 (4–8) |  | 5 (4–5) |  | 4 (3–6) |  | 14 (13–18) |  |
| No idea | 5 (4–7) |  | 4 (3–5) |  | 4 (3–5) |  | 14 (11–16) |  |
| **Received Information about T2D**** | | | | | | | | |
| Did not receive | 5 (4–6) | –4.54, <0.001 | 4 (3–5) | –5.23, <0.001 | 4 (3–5) | –1.57, 0.12 | 13 (11–16) | –5.66, <0.001 |
| Received | 5 (4–8) |  | 4 (3–5) |  | 4 (4–5) |  | 15 (12–17) |  |

*Spearman’s rank correlation

**Man–Whitney U test

***Kruskal–Wallis test
