# Supplementary material for: Knowledge, attitudes, and practices regarding type 2 diabetes and associated factors among rural adolescents in Indonesia: A cross-sectional study
Source: PLoS One. 2026 Jul 8;21(7):e0352982. doi: 10.1371/journal.pone.0352982 (PMC13345253; doi:10.1371/journal.pone.0352982)
Supplement: Attachment — Submitted filename: PROS ONE REVIEW REPORT.pdf [file pone.0352982.s006.pdf]

PLOS ONE REVIEW REPORT

MANUSCRIPT TITTLE: KNOWLEDGE, ATTITUDES AND PRACTICES REGARDING TYPE TWO DIABETES AND ASSOCIATED FACTORS AMONG RURAL ADOLESCENTS IN INDONESIA: A CROSS SECTIONAL STUDY

**SECTION**	**RECOMMENDATION**
Abstract is too dense and slightly inconsistent [Overloaded with numbers too many for statistics for readability]	Reduce statistical details; focus on only key findings and remove less critical regression coefficients
Interpretation of knowledge scores[manuscript reports a median knowledge score of 5/18 but does not provide framework for interpreting the value	Add interpretation scale-poor, moderate,good to contextualize the findings for readers
Limited scope of practice domain[you only included physical activity, smoking,and weight monitoring leaving out key behaviors such as diet,screen time.	Justify why these were excluded or acknowledge it in your study limitations
Use of linear regression with non normal data[You used multiple linear regression but KAP scores are ordinal/bounded	Justify why linear regression is appropriate or or consider generalized linear model or ordinal regression
Interpretation of effect size[B values are presented without practical interpretation]	Include brief explanation of what effect size represents in real terms and clarify whether observed differences are meaningful in practice
Causal language in cross sectional study[some statements imply causality which is not appropriate for cross sectional study]----longer school hours improve practice	Replace causal language with associative terms like associated with. Ensure consistence throughout your manuscript
Incomplete reporting requirements<br>data availability statement, funding statement, competing interest declaration are missing.	Include all required sections to comply with journal guidelines
Ethical considerations[Data handling and confidentiality measures are not detailed	Expand ethics section to include data anonymization and secure storage procedures
Language and grammar[some sentences are overly long and may reduce clarity.<br>Minor grammatical errors and typo issues are present]	Conduct thorough language editing for clarity and conciseness
Repetition [Some redundancy exists between introduction and discussion sections]	Reduce repetitive content to improve flow and readability
Theoretical framework[there is a brief reference on behavioral models without sufficient integration]	Expand discussion of relevant framework-health belief model and social cognitive theory to strengthen interpretation of knowledge gap

REVIEWERS NAME; SR ATUHAIRE PHIONAH
phionaatuhaire98@gmail.com
