# Supplementary material for: Knowledge, attitudes, and practices regarding type 2 diabetes and associated factors among rural adolescents in Indonesia: A cross-sectional study
Source: PLoS One. 2026 Jul 8;21(7):e0352982. doi: 10.1371/journal.pone.0352982 (PMC13345253; doi:10.1371/journal.pone.0352982)
Supplement: Attachment — Submitted filename: Response to Reviewers.docx [file pone.0352982.s008.docx]

**Response to Reviewers and Editors**

**Manuscript title:** *Knowledge, attitudes, and practices regarding type 2 diabetes and associated factors among rural adolescents in Indonesia: a cross-sectional study*

We sincerely thank the Editor and Reviewers for their insightful and constructive comments. We carefully revised the manuscript in response to all suggestions. Major revisions included clarification of statistical methods, expansion of ethical and reporting details, integration of behavioral frameworks, simplification of tables, addition of reporting statements and supporting files, and revision of language throughout the manuscript. All changes are reflected in the revised manuscript and supporting information.

**EDITOR’S COMMENTS**

**Comment 1:** Ensure that your manuscript meets PLOS ONE's style requirements, including those for file naming

**Response:**

We reviewed the manuscript and supporting files to ensure full compliance with PLOS ONE style requirements, including manuscript formatting, table presentation, supporting information organization, and file naming conventions.

**Formatting:**
The manuscript text was revised to conform to PLOS ONE formatting requirements, including heading structure, paragraph formatting, and table presentation.

**Table presentation:**
We revised the tables according to the journal guidelines. All tables were reformatted according to PLOS ONE guidelines and included as editable tables within the manuscript: Table 1 on pages 13–15, Table 2 on pages 15–16, Table 3 on page 17, Table 4 on page 18, Table 5 on page 19, Table 6 on pages 22–24, and Table 7 on pages 24–25.

**Supplementary material organization:**
Supporting files were appropriately cited in the manuscript text, including S1 File (page 8, line 168), S2 File (page 8, line 169), S3 File (page 10, line 237), S4 Checklist (page 11, line 240), and S5 Table (page 19, line 355).

**File naming conventions:**
We also revised the supporting information file names on page 38, lines 751–755, as follows:

- S1 File. Questionnaire in Indonesian language.
- S2 File. Questionnaire in English language.
- S3 File. Inclusivity in Global Research Questionnaire.
- S4 Checklist. STROBE checklist.
- S5 Table. Results of the bivariate analyses.

In addition, because our study used a cross-sectional design, we completed and included the STROBE checklist (S4 Checklist. STROBE checklist), in accordance with the journal guidelines. The checklist is cited in the Methods section on pages 10–11, lines 237–240, under the subtitle “Inclusivity in Global Research and Reporting Guidelines.”

**Comment 2:** Please include a complete copy of PLOS’ questionnaire on inclusivity in global research in your revised manuscript

**Response:**

We have included a complete copy of the PLOS inclusivity in global research questionnaire in the revised manuscript submission, as requested (S3 File. Inclusivity in Global Research Questionnaire; listed in the Supporting Information section on page 38, line 753).

We also added a subsection on inclusivity in global research in the Methods section under a separate subtitle, in accordance with the journal guidelines, on page 10, lines 235–237. In accordance with the PLOS inclusivity in global research guidance, we also added the following sentence to the Ethical Considerations section on page 10, lines 216–218: “The study was conducted in accordance with the approved protocol, and no major deviations occurred during the study period.”

**Comment 3:** If the reviewer comments include a recommendation to cite specific previously published works, please review and evaluate these publications to determine whether they are relevant and should be cited. There is no requirement to cite these works unless the editor has indicated otherwise.

**Response:**

We did not receive reviewer recommendations to cite additional references; therefore, no citations were included specifically in response to this point. However, we added two additional references to support and accommodate the revisions made throughout the manuscript.

**Comment 4:** Please review your reference list to ensure that it is complete and correct. If you have cited papers that have been retracted, please include the rationale for doing so in the manuscript text, or remove these references and replace them with relevant current references. Any changes to the reference list should be mentioned in the rebuttal letter that accompanies your revised manuscript. If you need to cite a retracted article, indicate the article’s retracted status in the References list and include a citation and full reference for the retraction notice.

**Response:**

We reviewed the reference list to ensure that all citations were complete, accurate, and current. We did not identify any retracted articles among the references cited in the manuscript.

**REVIEWER 1 COMMENTS**

**Comment 1:** The statistical analysis is comprehensive, but justification for using linear regression on non-normally distributed scores should be clarified.

**Response:**

We appreciate the reviewer’s comment. The non-normal distribution of KAP scores was reported primarily because non-parametric tests were used in the bivariate analyses.

Although the KAP scores were non-normally distributed and bounded by minimum and maximum values, they were derived as summed composite scores with multiple possible values and were therefore treated as approximately continuous outcomes for the regression analyses. Importantly, multiple linear regression does not require the dependent variable itself to be normally distributed; rather, the assumptions primarily concern the distribution of residuals and overall model fit. Diagnostic assessments indicated acceptable model fit and that residual assumptions were adequately satisfied. In addition, robust standard errors were applied to address potential heteroskedasticity.

We have clarified this rationale and the associated diagnostic assessments in the Methods section of the revised manuscript (Pages 11–12, lines 248–278). We also clarified the scoring procedures for the knowledge, attitude, practice, and overall KAP domains in the Methods section (Pages 8–9).

**Comment 2:** The discussion could better elaborate on practical intervention models tailored to rural school settings.

**Response:**

We have expanded the Discussion section to further elaborate on practical and contextually appropriate intervention models for rural Indonesian school settings, including school-based health education, peer-led approaches, extracurricular activities, and behavior-focused preventive strategies that may be feasible in resource-limited environments (Discussion section, pages 23–30).

**Comment 3:** Some tables (especially Table 5) are dense and may benefit from simplification for readability.

**Response:**

We have simplified Table 5, which is now presented as Table 6, to improve readability and clarity of presentation. Specifically, redundant statistical details were minimized, and the table layout was streamlined while retaining the key findings necessary for interpretation (Pages 22–24). The rows for the constant and model fit statistics were removed, and the model fit information along with other relevant notes are now presented in the table footnote (Page 24, lines 420–421).

**Comment 4:** Minor English language editing and proofreading are recommended to improve clarity and flow.

**Response:**

We revised the manuscript to improve clarity, readability, and overall flow. Additional English language editing and proofreading were conducted throughout the manuscript, including simplification of long sentences, refinement of grammar, and improvement of transitions between sections (Abstract, Introduction, Methods, and Discussion sections).

**REVIEWER 2 COMMENTS**

**Comment 1:** Abstract is too dense and slightly inconsistent [Overloaded with too many numbers for statistics for readability].

**Response**

We revised the abstract, particularly the Results section, to improve clarity and readability by reducing excessive statistical detail and removing less essential regression coefficients and p-values. The revised abstract now focuses more clearly on the main findings, key associations, and overall study implications while maintaining consistency with the full manuscript and ensuring that the abstract remains within the journal’s word limit (Page 2, lines 38–45).

**Comment 2.** Interpretation of knowledge scores [manuscript reports a median knowledge score of 5/18 but does not provide framework for interpreting the value.

**Response**

Thank you for this valuable feedback. Because median (IQR) values alone do not provide standardized interpretation of KAP levels, we incorporated modified Bloom’s cutoff criteria to classify KAP scores into low, moderate, and high categories. This approach was used to improve interpretability and provide clearer context for readers. We have clarified this classification approach in the Methods section (Page 11, lines 248–252) and incorporated the categorized findings into the Results section (Pages 18–19, lines 339–351, Table 5. Distribution of knowledge, attitude, practice and overall KAP).

**Comment 3.** Limited scope of practice domain [you only included physical activity, smoking, and weight monitoring leaving out key behaviors such as diet, screen time.

**Response**

We acknowledge that the practice domain did not include several relevant diabetes-related behaviors, such as dietary habits, screen time, and sleep patterns. To address this concern, we have added this point to the limitations section of the manuscript and clarified that the practice domain focused primarily on physical activity, smoking, and weight monitoring (Pages 31–32, lines 591–594).

**Comment 4.** Use of linear regression with non-normal data[You used multiple linear regression, but KAP scores are ordinal/bounded

**Response**

We appreciate the reviewer’s comment. The non-normal distribution of KAP scores was reported primarily because non-parametric tests were used in the bivariate analyses.

Although the KAP scores were non-normally distributed and bounded by minimum and maximum values, they were derived as summed composite scores with multiple possible values and were therefore treated as approximately continuous outcomes for the regression analyses. Importantly, multiple linear regression does not require the dependent variable itself to be normally distributed; rather, the assumptions primarily concern the distribution of residuals and overall model fit. Diagnostic assessments indicated acceptable model fit and that residual assumptions were adequately satisfied. In addition, robust standard errors were applied to address potential heteroskedasticity.

We have clarified this rationale and the associated diagnostic assessments in the Methods section of the revised manuscript (Pages 11–12, lines 248–268). We also clarified the scoring procedures for the knowledge, attitude, practice, and overall KAP domains in the Methods section (Pages 8–9)

**Comment 5.** Interpretation of effect size[B values are presented without practical interpretation]

**Response**

We revised the Results section, particularly the multivariable analysis of sociodemographic factors, to provide clearer interpretations of the regression coefficients. Instead of using only general associative language, we incorporated more specific interpretations, such as indicating higher or lower KAP scores in relation to key factors, alongside the reported statistical significance. Specifically, we clarified the approximate differences in KAP scores associated with important sociodemographic characteristics to help readers better understand the practical meaning of the observed effect sizes. We also used cautious and non-causal language throughout to avoid overstating the practical importance of the associations (Pages 20–22, lines 365–409). In addition, explanations regarding the practical interpretation of effect sizes and whether the observed differences may be meaningful in practice were added on page 22 (lines 410–415).

**Comment 6.** Causal language in cross sectional study[ some statements imply causality which is not appropriate for cross sectional study]----longer school hours improve practice.

**Response**

We carefully revised the manuscript to avoid causal language that may not be appropriate for a cross-sectional study design. Statements implying causality were replaced with associative terms such as “associated with,” “linked to,” or “may reflect,” and these revisions were applied consistently throughout the manuscript, including the Results, Discussion, and Conclusion sections.

**Comment 7.** Incomplete reporting requirements data availability statement, funding statement, competing interest declaration are missing.

**Response**

In the revised manuscript, we added a Data Availability Statement, Funding Statement, and Competing Interests Statement in accordance with PLOS ONE reporting requirements. The Data Availability Statement includes access to the study data through the Open Science Framework (OSF) repository (Page 33, lines 614–635). In addition, we included a completed STROBE checklist to improve reporting transparency and completeness (Pages 10–11, lines 237–240).

**Comment 8.** Ethical considerations[Data handling and confidentiality measures are not detailed

**Response**

We revised the Ethics section to provide clearer details regarding data handling and confidentiality procedures. Specifically, we clarified that questionnaires were anonymous, no identifying information was collected, completed questionnaires were coded and stored securely in a locked cabinet accessible only to the research team, and data were analyzed in aggregate form to maintain participant confidentiality. We also added information regarding the data retention period (Page 10, lines 228–233).

**Comment 9.** Language and grammar[some sentences are overly long and may reduce clarity. Minor grammatical errors and typo issues are present]

**Response**

We carefully revised the manuscript to improve readability and clarity. Several long and complex sentences were simplified, and sentence structures were refined throughout the manuscript to improve flow, reduce redundancy, and enhance comprehension while preserving the original meaning.

**Comment 10.** Repetition [Some redundancy exists between introduction and discussion sections]

**Response**

We carefully revised the manuscript to reduce redundancy between the Introduction and Discussion sections. Background information that overlapped with the Discussion was condensed or removed, and several sentences were simplified to improve readability and flow. The Introduction now focuses more clearly on the study rationale and research gaps, while the Discussion emphasizes interpretation of findings, comparison with previous studies, and implications for practice and policy.

**Comment 11.** Theoretical framework[there is a brief reference on behavioral models without sufficient integration]

**Response**

In the revised Discussion section, we expanded the theoretical interpretation of the observed knowledge–practice gap by further integrating relevant behavioral frameworks, particularly the Health Belief Model and Social Cognitive Theory. Specifically, we elaborated on how limited perceived susceptibility, insufficient cues to action, peer modeling, family influences, social reinforcement, and self-efficacy may help explain why awareness and positive attitudes did not consistently translate into healthier preventive practices among adolescents. These additions strengthen the interpretation of our findings and support the need for behaviorally informed school-based interventions (Page 30, lines 546–554).
