# Supplementary material for: Knowledge, attitudes, and practices regarding type 2 diabetes and associated factors among rural adolescents in Indonesia: A cross-sectional study
Source: PLoS One. 2026 Jul 8;21(7):e0352982. doi: 10.1371/journal.pone.0352982 (PMC13345253; doi:10.1371/journal.pone.0352982)
Supplement: Peer review history

# Peer review: Decision Letter 0

**DOI:** 10.1371/journal.pone.0352982.r001

**Authors:** Cho Lee Wong

20 May 2026

PONE-D-25-61411

Knowledge, attitudes, and practices regarding type 2 diabetes and associated factors among rural adolescents in Indonesia: a cross-sectional study

PLOS One

Dear Dr. Kusumawati,

Thank you for submitting your manuscript to PLOS ONE. After careful consideration, we feel that it has merit but does not fully meet PLOS ONE’s publication criteria as it currently stands. Therefore, we invite you to submit a revised version of the manuscript that addresses the points raised during the review process.

Please submit your revised manuscript by Jul 04 2026 11:59PM. If you will need more time than this to complete your revisions, please reply to this message or contact the journal office at plosone@plos.org. When you're ready to submit your revision, log on to https://www.editorialmanager.com/pone/ and select the 'Submissions Needing Revision' folder to locate your manuscript file.

Please include the following items when submitting your revised manuscript:

- A letter that responds to each point raised by the academic editor and reviewer(s). You should upload this letter as a separate file labeled 'Response to Reviewers'.
- A marked-up copy of your manuscript that highlights changes made to the original version. You should upload this as a separate file labeled 'Revised Manuscript with Track Changes'.
- An unmarked version of your revised paper without tracked changes. You should upload this as a separate file labeled 'Manuscript'.

If you would like to make changes to your financial disclosure, please include your updated statement in your cover letter. Guidelines for resubmitting your figure files are available below the reviewer comments at the end of this letter.

If applicable, we recommend that you deposit your laboratory protocols in protocols.io to enhance the reproducibility of your results. Protocols.io assigns your protocol its own identifier (DOI) so that it can be cited independently in the future. For instructions see: https://journals.plos.org/plosone/s/submission-guidelines#loc-laboratory-protocols. Additionally, PLOS ONE offers an option for publishing peer-reviewed Lab Protocol articles, which describe protocols hosted on protocols.io. Read more information on sharing protocols at https://plos.org/protocols?utm_medium=editorial-email&utm_source=authorletters&utm_campaign=protocols.

As the corresponding author, your ORCID iD is verified in the submission system and will appear in the published article. PLOS supports the use of ORCID, and we encourage all coauthors to register for an ORCID iD and use it as well. Please encourage your coauthors to verify their ORCID iD within the submission system before final acceptance, as unverified ORCID iDs will not appear in the published article. *Only*  the individual author can complete the verification step; PLOS staff *cannot*  verify ORCID iDs on behalf of authors.

We look forward to receiving your revised manuscript.

Kind regards,

Cho Lee Wong, PhD

Academic Editor

PLOS One

**Journal Requirements:**

When submitting your revision, we need you to address these additional requirements.

1. Please ensure that your manuscript meets PLOS ONE's style requirements, including those for file naming. The PLOS ONE style templates can be found at

https://journals.plos.org/plosone/s/file?id=wjVg/PLOSOne_formatting_sample_main_body.pdf and

https://journals.plos.org/plosone/s/file?id=ba62/PLOSOne_formatting_sample_title_authors_affiliations.pdf

2. Please include a complete copy of PLOS’ questionnaire on inclusivity in global research in your revised manuscript. Our policy for research in this area aims to improve transparency in the reporting of research performed outside of researchers’ own country or community. The policy applies to researchers who have travelled to a different country to conduct research, research with Indigenous populations or their lands, and research on cultural artefacts. The questionnaire can also be requested at the journal’s discretion for any other submissions, even if these conditions are not met.  Please find more information on the policy and a link to download a blank copy of the questionnaire here: https://journals.plos.org/plosone/s/best-practices-in-research-reporting. Please upload a completed version of your questionnaire as Supporting Information when you resubmit your manuscript.

3. If the reviewer comments include a recommendation to cite specific previously published works, please review and evaluate these publications to determine whether they are relevant and should be cited. There is no requirement to cite these works unless the editor has indicated otherwise.

4. Please review your reference list to ensure that it is complete and correct. If you have cited papers that have been retracted, please include the rationale for doing so in the manuscript text, or remove these references and replace them with relevant current references. Any changes to the reference list should be mentioned in the rebuttal letter that accompanies your revised manuscript. If you need to cite a retracted article, indicate the article’s retracted status in the References list and also include a citation and full reference for the retraction notice.

Reviewers' comments:

Reviewer's Responses to Questions

**Comments to the Author**

1. Is the manuscript technically sound, and do the data support the conclusions?

The manuscript must describe a technically sound piece of scientific research with data that supports the conclusions. Experiments must have been conducted rigorously, with appropriate controls, replication, and sample sizes. The conclusions must be drawn appropriately based on the data presented.

Reviewer #1: Yes

Reviewer #2: Yes

**********

2. Has the statistical analysis been performed appropriately and rigorously?

Reviewer #1: I Don't Know

Reviewer #2: No

**********

3. Have the authors made all data underlying the findings in their manuscript fully available?

The PLOS Data policy requires authors to make all data underlying the findings described in their manuscript fully available without restriction, with rare exception (please refer to the Data Availability Statement in the manuscript PDF file). The data should be provided as part of the manuscript or its supporting information, or deposited to a public repository. For example, in addition to summary statistics, the data points behind means, medians and variance measures should be available. If there are restrictions on publicly sharing data—e.g. participant privacy or use of data from a third party—those must be specified.

Reviewer #1: Yes

Reviewer #2: Yes

**********

4. Is the manuscript presented in an intelligible fashion and written in standard English?

PLOS ONE does not copyedit accepted manuscripts, so the language in submitted articles must be clear, correct, and unambiguous. Any typographical or grammatical errors should be corrected at revision, so please note any specific errors here.

Reviewer #1: No

Reviewer #2: No

**********

5. Review Comments to the Author

Please use the space provided to explain your answers to the questions above. You may also include additional comments for the author, including concerns about dual publication, research ethics, or publication ethics. (Please upload your review as an attachment if it exceeds 20,000 characters)

Reviewer #1: The manuscript addresses an important public health issue with a large and well-powered sample size.. The manuscript is really good. However, some recommendations are required:

The statistical analysis is comprehensive, but justification for using linear regression on non-normally distributed scores should be clarified.

The discussion could better elaborate on practical intervention models tailored to rural school settings.

Some tables (especially Table 5) are dense and may benefit from simplification for readability.

Minor English language editing and proofreading are recommended to improve clarity and flow.

Reviewer #2: Justify why linear regression is appropriate or or

consider generalized linear model or ordinal

regression

Include brief explanation of what effect size

represents in real terms and clarify whether

observed differences are meaningful in practice

Incomplete reporting requirements

data availability statement, funding statement,

competing interest declaration are missing.

Include all required sections to comply with

journal guidelines

**********

6. PLOS authors have the option to publish the peer review history of their article (what does this mean?). If published, this will include your full peer review and any attached files.

If you choose “no”, your identity will remain anonymous but your review may still be made public.

**Do you want your identity to be public for this peer review?**  For information about this choice, including consent withdrawal, please see our Privacy Policy.

Reviewer #1: **Yes:** Nasser M Alorfi

Reviewer #2: **Yes:** ATUHAIRE PHIONAH

**********

[NOTE: If reviewer comments were submitted as an attachment file, they will be attached to this email and accessible via the submission site. Please log into your account, locate the manuscript record, and check for the action link "View Attachments". If this link does not appear, there are no attachment files.]

To ensure your figures meet our technical requirements, please review our figure guidelines: https://journals.plos.org/plosone/s/figures

You may also use PLOS’s free figure tool, NAAS, to help you prepare publication quality figures: https://journals.plos.org/plosone/s/figures#loc-tools-for-figure-preparation.

NAAS will assess whether your figures meet our technical requirements by comparing each figure against our figure specifications.

Attachment Submitted filename: PROS ONE REVIEW REPORT.pdf

# Author comment: Author response to Decision Letter 1

**DOI:** 10.1371/journal.pone.0352982.r002

1 Jun 2026

Response to Reviewers and Editors

Manuscript title: Knowledge, attitudes, and practices regarding type 2 diabetes and associated factors among rural adolescents in Indonesia: a cross-sectional study

We sincerely thank the Editor and Reviewers for their insightful and constructive comments. We carefully revised the manuscript in response to all suggestions. Major revisions included clarification of statistical methods, expansion of ethical and reporting details, integration of behavioral frameworks, simplification of tables, addition of reporting statements and supporting files, and revision of language throughout the manuscript. All changes are reflected in the revised manuscript and supporting information.

EDITOR’S COMMENTS

Comment 1: Ensure that your manuscript meets PLOS ONE's style requirements, including those for file naming

Response:

We reviewed the manuscript and supporting files to ensure full compliance with PLOS ONE style requirements, including manuscript formatting, table presentation, supporting information organization, and file naming conventions.

Formatting:

The manuscript text was revised to conform to PLOS ONE formatting requirements, including heading structure, paragraph formatting, and table presentation.

Table presentation:

We revised the tables according to the journal guidelines. All tables were reformatted according to PLOS ONE guidelines and included as editable tables within the manuscript: Table 1 on pages 13–15, Table 2 on pages 15–16, Table 3 on page 17, Table 4 on page 18, Table 5 on page 19, Table 6 on pages 22–24, and Table 7 on pages 24–25.

Supplementary material organization:

Supporting files were appropriately cited in the manuscript text, including S1 File (page 8, line 168), S2 File (page 8, line 169), S3 File (page 10, line 237), S4 Checklist (page 11, line 240), and S5 Table (page 19, line 355).

File naming conventions:

We also revised the supporting information file names on page 38, lines 751–755, as follows:

• S1 File. Questionnaire in Indonesian language.

• S2 File. Questionnaire in English language.

• S3 File. Inclusivity in Global Research Questionnaire.

• S4 Checklist. STROBE checklist.

• S5 Table. Results of the bivariate analyses.

In addition, because our study used a cross-sectional design, we completed and included the STROBE checklist (S4 Checklist. STROBE checklist), in accordance with the journal guidelines. The checklist is cited in the Methods section on pages 10–11, lines 237–240, under the subtitle “Inclusivity in Global Research and Reporting Guidelines.”

Comment 2: Please include a complete copy of PLOS’ questionnaire on inclusivity in global research in your revised manuscript

Response:

We have included a complete copy of the PLOS inclusivity in global research questionnaire in the revised manuscript submission, as requested (S3 File. Inclusivity in Global Research Questionnaire; listed in the Supporting Information section on page 38, line 753).

We also added a subsection on inclusivity in global research in the Methods section under a separate subtitle, in accordance with the journal guidelines, on page 10, lines 235–237. In accordance with the PLOS inclusivity in global research guidance, we also added the following sentence to the Ethical Considerations section on page 10, lines 216–218: “The study was conducted in accordance with the approved protocol, and no major deviations occurred during the study period.”

Comment 3: If the reviewer comments include a recommendation to cite specific previously published works, please review and evaluate these publications to determine whether they are relevant and should be cited. There is no requirement to cite these works unless the editor has indicated otherwise.

Response:

We did not receive reviewer recommendations to cite additional references; therefore, no citations were included specifically in response to this point. However, we added two additional references to support and accommodate the revisions made throughout the manuscript.

Comment 4: Please review your reference list to ensure that it is complete and correct. If you have cited papers that have been retracted, please include the rationale for doing so in the manuscript text, or remove these references and replace them with relevant current references. Any changes to the reference list should be mentioned in the rebuttal letter that accompanies your revised manuscript. If you need to cite a retracted article, indicate the article’s retracted status in the References list and include a citation and full reference for the retraction notice.

Response:

We reviewed the reference list to ensure that all citations were complete, accurate, and current. We did not identify any retracted articles among the references cited in the manuscript.

REVIEWER 1 COMMENTS

Comment 1: The statistical analysis is comprehensive, but justification for using linear regression on non-normally distributed scores should be clarified.

Response:

We appreciate the reviewer’s comment. The non-normal distribution of KAP scores was reported primarily because non-parametric tests were used in the bivariate analyses.

Although the KAP scores were non-normally distributed and bounded by minimum and maximum values, they were derived as summed composite scores with multiple possible values and were therefore treated as approximately continuous outcomes for the regression analyses. Importantly, multiple linear regression does not require the dependent variable itself to be normally distributed; rather, the assumptions primarily concern the distribution of residuals and overall model fit. Diagnostic assessments indicated acceptable model fit and that residual assumptions were adequately satisfied. In addition, robust standard errors were applied to address potential heteroskedasticity.

We have clarified this rationale and the associated diagnostic assessments in the Methods section of the revised manuscript (Pages 11–12, lines 248–278). We also clarified the scoring procedures for the knowledge, attitude, practice, and overall KAP domains in the Methods section (Pages 8–9).

Comment 2: The discussion could better elaborate on practical intervention models tailored to rural school settings.

Response:

We have expanded the Discussion section to further elaborate on practical and contextually appropriate intervention models for rural Indonesian school settings, including school-based health education, peer-led approaches, extracurricular activities, and behavior-focused preventive strategies that may be feasible in resource-limited environments (Discussion section, pages 23–30).

Comment 3: Some tables (especially Table 5) are dense and may benefit from simplification for readability.

Response:

We have simplified Table 5, which is now presented as Table 6, to improve readability and clarity of presentation. Specifically, redundant statistical details were minimized, and the table layout was streamlined while retaining the key findings necessary for interpretation (Pages 22–24). The rows for the constant and model fit statistics were removed, and the model fit information along with other relevant notes are now presented in the table footnote (Page 24, lines 420–421).

Comment 4: Minor English language editing and proofreading are recommended to improve clarity and flow.

Response:

We revised the manuscript to improve clarity, readability, and overall flow. Additional English language editing and proofreading were conducted throughout the manuscript, including simplification of long sentences, refinement of grammar, and improvement of transitions between sections (Abstract, Introduction, Methods, and Discussion sections).

REVIEWER 2 COMMENTS

Comment 1: Abstract is too dense and slightly inconsistent [Overloaded with too many numbers for statistics for readability].

Response

We revised the abstract, particularly the Results section, to improve clarity and readability by reducing excessive statistical detail and removing less essential regression coefficients and p-values. The revised abstract now focuses more clearly on the main findings, key associations, and overall study implications while maintaining consistency with the full manuscript and ensuring that the abstract remains within the journal’s word limit (Page 2, lines 38–45).

Comment 2. Interpretation of knowledge scores [manuscript reports a median knowledge score of 5/18 but does not provide framework for interpreting the value.

Response

Thank you for this valuable feedback. Because median (IQR) values alone do not provide standardized interpretation of KAP levels, we incorporated modified Bloom’s cutoff criteria to classify KAP scores into low, moderate, and high categories. This approach was used to improve interpretability and provide clearer context for readers. We have clarified this classification approach in the Methods section (Page 11, lines 248–252) and incorporated the categorized findings into the Results section (Pages 18–19, lines 339–351, Table 5. Distribution of knowledge, attitude, practice and overall KAP).

Comment 3. Limited scope of practice domain [you only included physical activity, smoking, and weight monitoring leaving out key behaviors such as diet, screen time.

Response

We acknowledge that the practice domain did not include several relevant diabetes-related behaviors, such as dietary habits, screen time, and sleep patterns. To address this concern, we have added this point to the limitations section of the manuscript and clarified that the practice domain focused primarily on physical activity, smoking, and weight monitoring (Pages 31–32, lines 591–594).

Comment 4. Use of linear regression with non-normal data[You used multiple linear regression, but KAP scores are ordinal/bounded

Response

We appreciate the reviewer’s comment. The non-normal distribution of KAP scores was reported primarily because non-parametric tests were used in the bivariate analyses.

Although the KAP scores were non-normally distributed and bounded by minimum and maximum values, they were derived as summed composite scores with multiple possible values and were therefore treated as approximately continuous outcomes for the regression analyses. Importantly, multiple linear regression does not require the dependent variable itself to be normally distributed; rather, the assumptions primarily concern the distribution of residuals and overall model fit. Diagnostic assessments indicated acceptable model fit and that residual assumptions were adequately satisfied. In addition, robust standard errors were applied to address potential heteroskedasticity.

We have clarified this rationale and the associated diagnostic assessments in the Methods section of the revised manuscript (Pages 11–12, lines 248–268). We also clarified the scoring procedures for the knowledge, attitude, practice, and overall KAP domains in the Methods section (Pages 8–9)

Comment 5. Interpretation of effect size[B values are presented without practical interpretation]

Response

We revised the Results section, particularly the multivariable analysis of sociodemographic factors, to provide clearer interpretations of the regression coefficients. Instead of using only general associative language, we incorporated more specific interpretations, such as indicating higher or lower KAP scores in relation to key factors, alongside the reported statistical significance. Specifically, we clarified the approximate differences in KAP scores associated with important sociodemographic characteristics to help readers better understand the practical meaning of the observed effect sizes. We also used cautious and non-causal language throughout to avoid overstating the practical importance of the associations (Pages 20–22, lines 365–409). In addition, explanations regarding the practical interpretation of effect sizes and whether the observed differences may be meaningful in practice were added on page 22 (lines 410–415).

Comment 6. Causal language in cross sectional study[ some statements imply causality which is not appropriate for cross sectional study]----longer school hours improve practice.

Response

We carefully revised the manuscript to avoid causal language that may not be appropriate for a cross-sectional study design. Statements implying causality were replaced with associative terms such as “associated with,” “linked to,” or “may reflect,” and these revisions were applied consistently throughout the manuscript, including the Results, Discussion, and Conclusion sections.

Comment 7. Incomplete reporting requirements data availability statement, funding statement, competing interest declaration are missing.

Response

In the revised manuscript, we added a Data Availability Statement, Funding Statement, and Competing Interests Statement in accordance with PLOS ONE reporting requirements. The Data Availability Statement includes access to the study data through the Open Science Framework (OSF) repository (Page 33, lines 614–635). In addition, we included a completed STROBE checklist to improve reporting transparency and completeness (Pages 10–11, lines 237–240).

Comment 8. Ethical considerations[Data handling and confidentiality measures are not detailed

Response

We revised the Ethics section to provide clearer details regarding data handling and confidentiality procedures. Specifically, we clarified that questionnaires were anonymous, no identifying information was collected, completed questionnaires were coded and stored securely in a locked cabinet accessible only to the research team, and data were analyzed in aggregate form to maintain participant confidentiality. We also added information regarding the data retention period (Page 10, lines 228–233).

Comment 9. Language and grammar[some sentences are overly long and may reduce clarity. Minor grammatical errors and typo issues are present]

Response

We carefully revised the manuscript to improve readability and clarity. Several long and complex sentences were simplified, and sentence structures were refined throughout the manuscript to improve flow, reduce redundancy, and enhance comprehension while preserving the original meaning.

Comment 10. Repetition [Some redundancy exists between introduction and discussion sections]

Response

We carefully revised the manuscript to reduce redundancy between the Introduction and Discussion sections. Background information that overlapped with the Discussion was condensed or removed, and several sentences were simplified to improve readability and flow. The Introduction now focuses more clearly on the study rationale and research gaps, while the Discussion emphasizes interpretation of findings, comparison with previous studies, and implications for practice and policy.

Comment 11. Theoretical framework[there is a brief reference on behavioral models without sufficient integration]

Response

In the revised Discussion section, we expanded the theoretical interpretation of the observed knowledge–practice gap by further integrating relevant behavioral frameworks, particularly the Health Belief Model and Social Cognitive Theory. Specifically, we elaborated on how limited perceived susceptibility, insufficient cues to action, peer modeling, family influences, social reinforcement, and self-efficacy may help explain why awareness and positive attitudes did not consistently translate into healthier preventive practices among adolescents. These additions strengthen the interpretation of our findings and support the need for behaviorally informed school-based interventions (Page 30, lines 546–554).

Attachment Submitted filename: Response to Reviewers.docx

# Editor report: Decision Letter 1

**DOI:** 10.1371/journal.pone.0352982.r003

**Authors:** Cho Lee Wong, Cho Lee Wong

18 Jun 2026

Knowledge, attitudes, and practices regarding type 2 diabetes and associated factors among rural adolescents in Indonesia: a cross-sectional study

PONE-D-25-61411R1

Dear Dr. Kusumawati,

We’re pleased to inform you that your manuscript has been judged scientifically suitable for publication and will be formally accepted for publication once it meets all outstanding technical requirements.

Within one week, you’ll receive an e-mail detailing the required amendments. When these have been addressed, you’ll receive a formal acceptance letter and your manuscript will be scheduled for publication.

An invoice will be generated when your article is formally accepted. Please note, if your institution has a publishing partnership with PLOS and your article meets the relevant criteria, all or part of your publication costs will be covered. Please make sure your user information is up-to-date by logging into Editorial Manager at Editorial Manager® and clicking the ‘Update My Information' link at the top of the page. For questions related to billing, please contact billing support.

If your institution or institutions have a press office, please notify them about your upcoming paper to help maximize its impact. If they’ll be preparing press materials, please inform our press team as soon as possible -- no later than 48 hours after receiving the formal acceptance. Your manuscript will remain under strict press embargo until 2 pm Eastern Time on the date of publication. For more information, please contact onepress@plos.org.

Kind regards,

Cho Lee Wong, PhD

Academic Editor

PLOS One

Additional Editor Comments (optional):

Reviewers' comments:

# Editor report: Acceptance letter

**DOI:** 10.1371/journal.pone.0352982.r004

**Authors:** Cho Lee Wong, Cho Lee Wong

PONE-D-25-61411R1

PLOS One

Dear Dr. Kusumawati,

I'm pleased to inform you that your manuscript has been deemed suitable for publication in PLOS One. Congratulations! Your manuscript is now being handed over to our production team.

At this stage, our production department will prepare your paper for publication. This includes ensuring the following:

* All references, tables, and figures are properly cited

* All relevant supporting information is included in the manuscript submission,

* There are no issues that prevent the paper from being properly typeset

You will receive further instructions from the production team, including instructions on how to review your proof when it is ready. Please keep in mind that we are working through a large volume of accepted articles, so please give us a few days to review your paper and let you know the next and final steps.

Lastly, if your institution or institutions have a press office, please let them know about your upcoming paper now to help maximize its impact. If they'll be preparing press materials, please inform our press team within the next 48 hours. Your manuscript will remain under strict press embargo until 2 pm Eastern Time on the date of publication. For more information, please contact onepress@plos.org.

You will receive an invoice from PLOS for your publication fee after your manuscript has reached the completed accept phase. If you receive an email requesting payment before acceptance or for any other service, this may be a phishing scheme. Learn how to identify phishing emails and protect your accounts at https://explore.plos.org/phishing.

If we can help with anything else, please email us at customercare@plos.org.

Thank you for submitting your work to PLOS ONE and supporting open access.

Kind regards,

PLOS ONE Editorial Office Staff

on behalf of

Dr. Cho Lee Wong

Academic Editor

PLOS One
